# Unraveling *cis* and *trans* regulatory evolution during cotton domestication

**DOI:** 10.1038/s41467-019-13386-w

**Published:** 2019-11-27

**Authors:** Ying Bao, Guanjing Hu, Corrinne E. Grover, Justin Conover, Daojun Yuan, Jonathan F. Wendel

**Affiliations:** 1 School of Life Sciences, Qufu Normal University, 273165 Qufu, Shandong Province China; 2 Department of Ecology, Evolution and Organismal Biology, Iowa State University, Ames, IA 50011 USA

**Keywords:** Evolutionary genetics, Evolutionary biology, Polyploidy in plants

## Abstract

*Cis* and *trans* regulatory divergence underlies phenotypic and evolutionary diversification. Relatively little is understood about the complexity of regulatory evolution accompanying crop domestication, particularly for polyploid plants. Here, we compare the fiber transcriptomes between wild and domesticated cotton (*Gossypium hirsutum*) and their reciprocal F_1_ hybrids, revealing genome-wide (~15%) and often compensatory *cis* and *trans* regulatory changes under divergence and domestication. The high level of *trans* evolution (54%–64%) observed is likely enabled by genomic redundancy following polyploidy. Our results reveal that regulatory variation is significantly associated with sequence evolution, inheritance of parental expression patterns, co-expression gene network properties, and genomic loci responsible for domestication traits. With respect to regulatory evolution, the two subgenomes of allotetraploid cotton are often uncoupled. Overall, our work underscores the complexity of regulatory evolution during fiber domestication and may facilitate new approaches for improving cotton and other polyploid plants.

## Abstract (web summary)

Relatively little is known about the complexity of regulatory evolution accompanying polyploid crop domestication. Here, using reciprocal hybrids between wild and domesticated allotetraploid cotton lines, the authors catalog *cis* and *trans* regulatory variants and show their equivalent effects on cotton fiber domestication.

## Introduction

Since the time of Charles Darwin and Gregor Mendel, domesticated plants have been profoundly important for studying the genetic nature of phenotype divergence and evolution^1–3^. Through contrasts of plant morphology between cultivated crops and their wild progenitors, the consequences of selection may be studied in a context where the directionality of evolutionary change as well as timeframe are reasonably well understood, thus providing a powerful framework for revealing the genetic underpinnings of phenotypic change^3^. Molecular genetic experiments, often combined with fine-mapping and, more recently, comparative population genomics approaches, have led to significant progress in identifying causative genetic changes that underlie domestication traits. Collectively, these studies provide multiple examples of specific coding sequence variants (SNP/indel polymorphisms, amino acid substitutions, mis-splice mutations, transposon insertions) that have increased in frequency or been fixed by strong directional human selection^4–6^. In addition, these and other studies have illuminated the key role of gene regulation in generating new phenotypes^7,8^. One spectacular example is the *teosinte branched 1* (*tb1*) gene in maize, where selection (from standing variation) for overexpression, mediated by a transposon insertion, caused an increase in apical dominance during domestication of maize from its ancestor, teosinte^9^. This and other examples highlight the prominence of regulatory evolution in the origin of new phenotypes under domestication, and by extension, in natural settings as well^10,11^.

Gene expression is regulated through the interactions of *cis* and *trans* regulatory elements. *Cis* regulatory elements are short regions of DNA (typically non-coding sequences adjacent to coding regions) that comprise specific binding sites for *trans* acting factors (e.g. transcription factors); together, these elements control expression of their associated gene(s)^12^. Relative to genetic mutations in coding genes themselves, *cis* and *trans* variants modify phenotypes by introducing spatiotemporal polymorphisms in gene expression. This variation may be challenging to observe or quantify, because different genomic and/or cellular environments among samples may confound the contribution of *cis* and *trans* effects, particularly in non-homogeneous backgrounds. Accordingly, the first-generation intra- and inter-specific hybrids have been used for analyses of allele-specific expression (ASE), which isolate expression changes attributed to *cis* regulatory divergence between the parents, thereby facilitating observation of *trans* effects that are shared in the hybrid^13^. In plants, ASE analyses have showed abundant *cis* regulatory divergence both within^14,15^ and between species^16–18^ at steady states, as well as in response to abiotic stresses^19–21^. However, the question of how directional human-mediated selection under domestication affects *cis* vs. *trans* regulatory evolution remains under-explored, a notable exception being the canonical example from maize^16^.

Cotton (*Gossypium*) ranks among the most important agronomic genera in the world, providing the foundation of the natural textile and for its value as an oil seed. Four cotton species, including two American tetraploids (*G. hirsutum* and *G. barbadense*) and two African-Asian diploids (*G. arboreum* and *G. herbaceum*), were independently domesticated within the last 8000 years^22^, and of these, upland cotton (*G. hirsutum* L.) presently accounts for more than 90% of cotton fiber production globally. *Gossypium hirsutum* is an allotetraploid species (AD genome), containing two largely collinear genomes (A and D) donated by it diploid progenitors 1–2 million years ago^23^. One interesting and relevant feature of the allopolyploid genome is that its two subgenomes differ two-fold in size despite having approximately the same number of genes; this raises the prospect that polyploidy was accompanied by the merger of two rather different *cis/trans* regulatory systems, which subsequently have been shaped by natural evolution^24^. Understanding *cis and* trans interactions in an allopolyploid is not only made more complicated by the presence of duplicated suites of interacting factors, but by the fact that even in a diploid regulatory interactions are subject to many different forms of biochemical and stoichiometric control and feedbacks^25^.

**Fig. 1:**
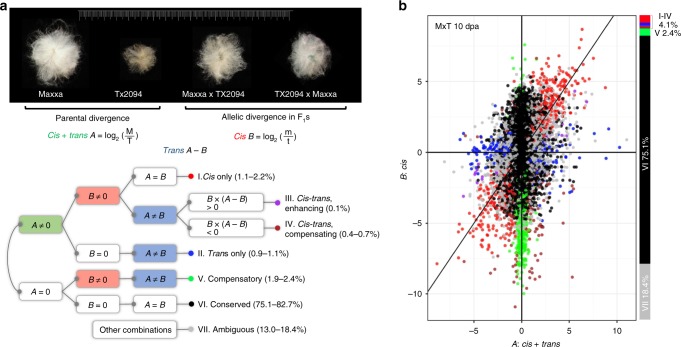
Parental and F_1_ hybrid expression data enable the ASE study of *G. hirsutum* domestication. **a** Using parental and F_1_ allelic expression divergence between wild and domesticated cottons, genes were assigned into one of seven regulatory categories representing combinations of *cis* and *trans* regulatory effects. Briefly, differential expression of any given gene in the parents reflects both *cis* and *trans* divergence (***A***), whereas the expression of the same gene in the common *trans* environment of the F_1_ hybrid reflects *cis* regulatory divergence (***B***) between parental alleles. While *trans* divergence cannot be directly measured, it can be inferred via the difference in ***A*** − ***B***. See methods for additional description. Next to each category, the percentage range of genes was obtained from four sample conditions (M × T and T × M hybrids each at 10 and 20 dpa). **b** Taking the 10 dpa sample of the F_1_ hybrid M × T as an example, the scatter plot of *cis* regulatory divergence (*y*-axis) vs. parental expression divergence (x-axis) is shown for all seven categories of genes. Category I–IV together only account for 4.1% of 27,816 genes, with the majority of genes assigned to categories of conserved (VI–75.1%), ambiguous (VII–18.4%), or compensatory regulation (V–2.4%). The source data underlying Fig. 1b are provided as a Source Data file.

Despite the striking changes involved in cotton domestication, transforming fibers (single celled seed trichomes) from light brown, short (<1 cm) and tightly adherent, to white, long (up to 5 cm) and easily removed (Fig. 1a, top), no domestication genes with major effects have been identified from QTL and whole-genome resequencing studies^4,26–28^. Also, little is known regarding the regulatory control and evolution of the complex genetic architecture of cotton fiber development^29^, nor of the massive expression changes caused by domestication^30–32^. Here we use reciprocal F_1_ hybrids between cultivated and wild accessions of allotetraploid cotton *G. hirsutum* to distinguish *cis* and *trans* effects accompanying cotton domestication. In addition to evaluating their relative contributions, we examine the inheritance of regulatory variants, their association with promoter sequence divergence, and how regulatory evolution shapes duplicated gene expression. We further explore the potential functions of relevant genes to fiber morphology in the context of gene co-expression and regulatory networks.

## Results

### Allelic expression analysis of cotton fiber transcriptomes

A total of 27 fiber RNA-seq libraries were sequenced, consisting of two timepoints each from the *G. hirsutum* cultivar Acala Maxxa (four replicates), the wild race Yucatanense TX2094 (3-4 replicates), and their reciprocal F_1_ hybrids (three replicates each) Maxxa × TX2094 (M × T) and TX2094 × Maxxa (T × M; Fig. 1a; Supplementary Table 1). To distinguish Maxxa and TX2094 alleles in the F_1_ hybrids, parental reads were used to generate a diagnostic SNP table, which comprised 91,017 SNPs distributed over 27,816 gene models (13,462 A-genome homoeologs, 14,060 D-genome homoeologs, and 294 on scaffolds without chromosome designation). From a total of 150 million F_1_ reads mapped to the reference genome, 7.5% and 7.7% account for allele-specific reads that were unambiguously assigned to Maxxa and TX2094 alleles, respectively. These proportions are consistent across the twelve F_1_ samples and between the reciprocal hybrids M × T and T × M (Supplementary Table 2). Thus, more TX2094-specific reads than Maxxa-specific reads were recovered, probably due to a higher level of heterozygosity in the former accession. This bias was corrected by read count normalization when calculating allelic expression levels in following analyses.

### Categorization of regulatory divergence under domestication

In Maxxa versus TX2094 comparisons, 6883 and 4829 genes were differentially expressed at two key fiber developmental stages, i.e., 10 and 20 days post-anthesis (dpa), respectively; the union of these accounted for 15% of the fiber transcriptome in *G. hirsutum*. As illustrated in Fig. 1, these expression changes under domestication, as measured by the log_2_ ratio of Maxxa:TX2094 parental read counts (***A***), reflect a combination of *cis-* and *trans-*regulatory effects. Under the common *trans* environment of the F_1_ hybrids, we were able to measure *cis* effects as the log_2_ ratio of Maxxa to TX2094 allele-specific read counts (***B***), for the 27,816 genes containing allelic SNPs in the transcript sequences. Accordingly, *trans* effects were measured as the difference between the parental and F_1_ expression divergence (***A***-***B***). When the same ASE measurements were used for ***B*** and subsequently ***A***-***B***, a strong negative correlation was found between the *cis* and *trans* effects with Pearson’s *r* = −0.78 to −0.80 (Supplementary Data 1). As recently noted^33^, this standard method is intrinsically biased and overestimates negative correlation, but alternatively, a cross-replicate approach can eliminate this bias by using different biological replicates for estimating *cis* and *trans* effects separately (see Methods). The resulting correlations (*r* = −0.29 to −0.34, *P* < 0.05; Supplementary Data 1) were weaker than those from the standard method, but remained significant.

**Fig. 2:**
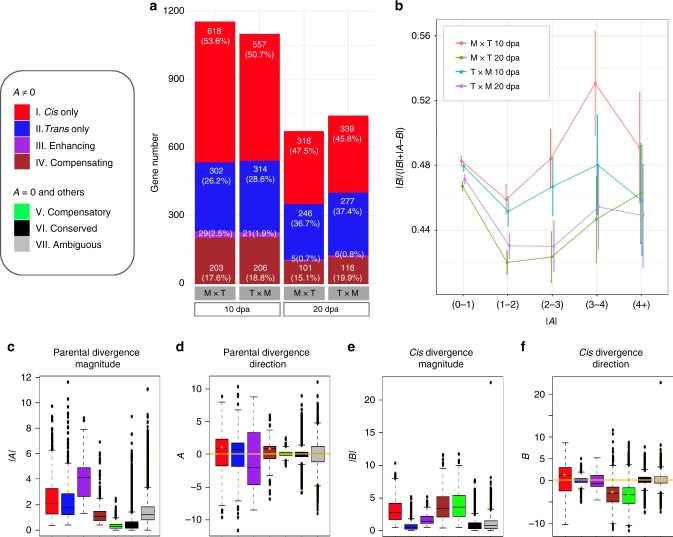
Categorization of *cis* and *trans* regulatory divergence. **a** Regulatory categories I–IV that exhibited parental divergence (***A*** ≠ 0). In 10 and 20 dpa fibers from the reciprocal F_1_ hybrids M × T and T × M, gene numbers and relative percentages of these four categories were shown. **b** Proportion of *cis* regulatory contribution to parental expression divergence. Genes were binned by absolute parental divergence |***A***| in *x*-axis, and the amount of absolute total expression divergence due to *cis* effects |***B***|/(|***B***| + |***A***−***B***|) is shown on the *y*-axis with error bars depicting 95% confidence intervals. **c**–**f** Boxplots showing the magnitude and direction of parental expression divergence and *cis* regulatory divergence, as summarized by pooled M × T and T × M data at both the 10 and 20 dpa developmental stages. Boxplot elements: center line–median; box limits–upper (Q3) and lower (Q1) quartiles; whiskers–smallest and largest non-outlier; points–outliers. *Y*-axis values above zero in **d**, **f** indicate a bias toward higher parental and allelic Maxxa expression, respectively; similarly, below zero indicates a bias towards TX2094. The significant deviations from zero, as indicated by gold star symbol (*), was inferred by Student’s *t*-test (*P* < 0.05). Corresponding plots for each F_1_ hybrid at either stage are shown in Supplementary Fig. 2. Source data are provided as a Source Data file.

Based on the statistical tests of ***A***, ***B***, and ***A*** vs. ***B***, genes were assigned into one of seven regulatory categories (Fig. 1a). In all four F_1_ samples (M × T and T × M each at 10 and 20 dpa), over 90% genes were classified into the categories of conserved (VI) and ambiguous (VII) expression. Of genes that exhibited parental expression divergence (***A*** ≠ 0, categories I to IV; Fig. 2a), ~80% were characterized as resulting from variation acting in *cis* only (I) or in *trans* only (II), where a higher proportion of I than II were consistently observed (Figs. 1a and 2a). When *cis* and *trans* effects were both detected, genes were further divided based on how regulatory variants each contributed to expression changes, in terms of their direction of action. The number of genes exhibiting opposite *cis* and *trans* effects (compensating; category IV) was 7- to 20-fold greater than those exhibiting both effects in the same direction (enhancing; category III). This excess of *cis* and *trans* changes acting in opposition was also evidenced by the genes exhibiting compensatory regulation (category V, Fig. 1a), when no differential expression was found between the parents, yet differential allelic expression was inferred in the F_1_ hybrids. Thus, the *cis* and *trans* variants in the parents operate antagonistically to produce similar expression (i.e., they are compensatory). This category is the third largest (Fig. 1b) following the conserved and ambiguous categories, regardless of the stringency of corresponding statistical tests (see Methods). Together with the negative correlations between the *cis* and *trans* effects directly measured above, these observations suggested that the antagonistic action of *cis* and *trans* effects is far more common than reinforcing changes, consistent with previous ASE studies^34^.

We next asked whether the crossing direction of hybridization has effects on the inference of regulatory divergence. Because the measure of ***A*** was common for M × T and T × M hybrids while each of their ***B*** values was separately inferred, the ASE inference was not completely independent between the reciprocal hybrids. We note that over 93% of genes (26,041 and 26,106 of 27,816 genes at 10 and 20 dpa, respectively) were consistently assigned into the same regulatory categories in the two reciprocal F_1_s. Excluding those genes exhibiting ambiguous regulation due to statistical incongruence in either F_1_ hybrid, this overlap increased to over 99% (Supplementary Fig. 1). Thus, the overlapping genes assigned to categories I to V each at 10 and 20 dpa (1321 and 923 genes, respectively) were considered to exhibit regulatory divergence (RD) under divergence and domestication, and their union resulted in a list of 1655 RD genes. Of these, 513 genes exhibited *cis* only divergence, 301 genes exhibited *trans* only divergence, and the remaining 841 genes exhibited both *cis* and *trans* divergence (Supplementary Data 2).

### *Cis* and *trans* contributions to expression divergence

Despite the higher numbers of *cis* only (I) versus *trans* only (II) genes (Fig. 2a), less than half regulatory divergence between TX2094 and Maxxa was due to *cis* effects, as measured by their relative contribution when considering all genes (standard method 46–48%, cross-replicate 36–45%; Supplementary Data 1). A significantly higher overall magnitude of *trans* than *cis* regulatory divergence was consistently found (|*trans*| > |*cis*|, Wilcoxon rank sum test *P* < 0.05; Supplementary Data 1). In addition, overall *trans* regulatory divergence correlated more highly with expression differences than did *cis* regulatory divergence (Pearson’s correlation *r* = 0.36–0.51 for *trans*, *r* = 0.17–0.27 for *cis*; Supplementary Data 1). Although the *cis* proportions appear to increase with the magnitude of parental expression divergence (absolute log_2_(M/T) ratios from 1 to 4), the Pearson correlations were rather weak (*r* = −0.02 to −0.08, *P* < 0.01; Fig. 2b).

Corresponding to the magnitude of parental expression divergence, as calculated by |***A***| (Fig. 2c; also see Supplementary Fig. 2 for each F_1_ hybrid at either fiber stage), the category of enhancing regulation exhibited the highest level of expression changes, followed by the *cis* only and *trans* only categories (III > I > II; Wilcoxon rank sum test, *P* < 0.05). Thus, when *cis* and *trans* effects acted in the same direction, the highest magnitude of parental expression change was observed, as may be expected. With respect to the direction of regulatory change, as calculated by ***A*** **(**Fig. 2d), categories of *cis* only and compensating regulation were biased toward up-regulating gene expression in Maxxa versus TX2094 (***A*** > 0 for I and IV, Student’s *t*-test *P* < 0.05), with no significant directional bias detected for other categories. Considering all categories together, 2715 and 1916 genes exhibited ***A*** > 0 at 10 and 20 dpa, significantly higher than the 2453 and 1612 genes that exhibited ***A*** ***<*** 0, respectively (Supplementary Table 3; Chi-squared test *P* < 0.05).

Because the magnitude of *cis* divergence in F_1_ hybrids is defined and estimated by |***B***| (Fig. 2e), values above zero were observed for genes assigned to categories of *cis* only, enhancing, compensating and compensatory regulation; as expected, *trans* only genes exhibited near zero magnitude of *cis* effect. In comparison with the compensating and compensatory regulation, a much lower level was found for the category of enhancing regulation (IV or V > III; Wilcoxon rank sum test *P* < 0.05). Regarding the direction of *cis* divergence (Fig. 2f), higher expression of Maxxa alleles was preferentially observed for *cis* only genes (***B*** > 0, Student’s *t*-test *P* < 0.05), consistent with the pattern of their parental divergence shown in Fig. 2d (***A*** > 0). In contrast, genes categorized as displaying antagonistic *ci*s and *trans* regulation exhibited lower expression of Maxxa alleles than TX2094 alleles (***B*** < 0 for IV and V, Student’s *t*-test *P* < 0.05). Considering all categories together, a significant bias toward lower expression of Maxxa alleles was observed with approximately twice as many ***B*** < 0 genes than ***B*** > 0 genes (Supplementary Table 3; Chi-squared test *P* < 0.05). The foregoing results collectively suggest that *cis* evolutionary change was biased toward favoring lower expression during domestication (***B*** < 0), whereas *trans* evolution acted antagonistically even to a larger extent to reverse this directional bias (***A*** > 0).

### Regulatory divergence is associated with mode of inheritance

**Fig. 3:**
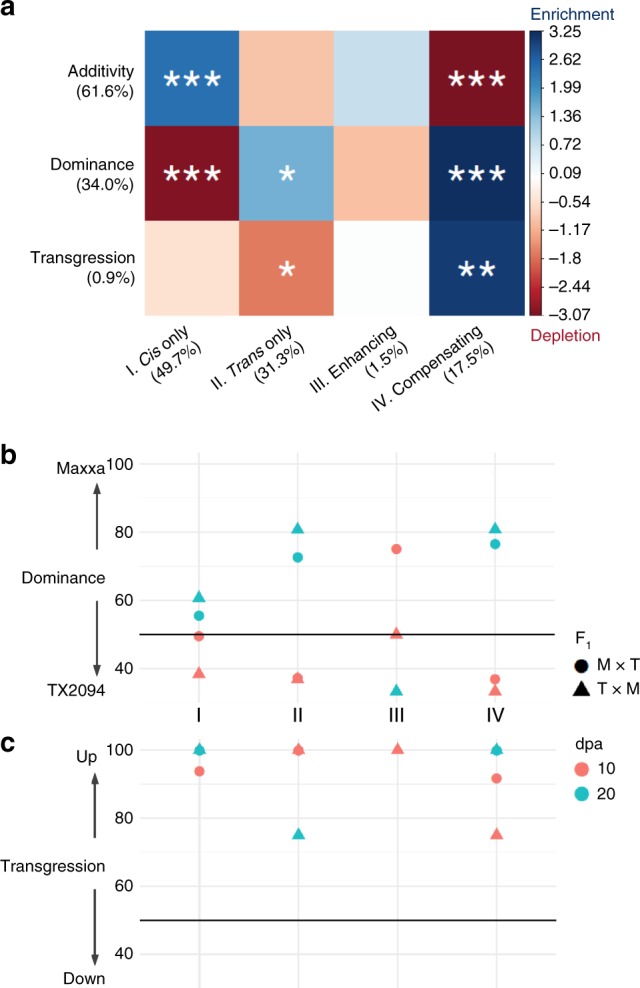
Relationships between regulatory mechanism and mode of inheritance. **a** For each combination of inheritance (rows) and *cis*/*trans* regulatory (columns) categories, the color shows the magnitude of gene enrichment (blue) and depletion (red) based on residuals of Pearson’s Chi-square test of independence. Blue indicates a positive residual when more genes were observed than expected under the null model of independence, and red indicates fewer genes than expected. Statistical significance was derived from Fisher’s exact test, as indicated by *P < 0.05, **P < 0.01, ***P < 0.001. **b** Within the category of dominance, percentages of Maxxa-dominant genes are shown for each regulatory category on the *x*-axis. **c** Within the category of transgression, the percentage of over-expressed genes are shown for each regulatory category on the *x*-axis. Source data are provided as a Source Data file.

To investigate the inheritance patterns of regulatory divergence under domestication, we classified the genes that exhibited parental expression change (***A*** ≠ 0) into the following inheritance categories: additive (where expression in the F_1_ hybrid is equivalent to the average of that of the two parents), dominant (where expression in the hybrid is equivalent to the expression of only one parent), and transgressive (where expression in the hybrid is outside the range of the two parental expression values). Overall, the majority of genes showed additive and dominant expression inheritance patterns (62% and 34%, respectively), with 1% of genes exhibiting transgressive up- or down-regulated expression, leaving about 3% that could not be assigned due to statistically conflicting patterns (Fig. 3a). A significant association was found between regulatory mechanism and the mode of expression inheritance (Chi-square test of independence, *P* < 0.05), with *cis* only regulation being enriched for additive inheritance and depleted of dominance, while both *trans* only and compensating regulation were enriched for genes that displayed dominance in their regulatory evolution. Interestingly, we observed a significant enrichment of transgressive inheritance in those genes exhibiting compensating regulation. No significant correspondence was detected for genes exhibiting enhancing *cis* and *trans* regulation probably due to the small size of this category.

Within the inheritance category of dominant expression, genes were further divided into patterns of Maxxa-dominant and TX2094-dominant, defined as whether the F_1_ expression is equivalent to the expression of Maxxa or TX2094, respectively. If there is no preference of either pattern, a 50:50 proportion of gene numbers is expected. In 20 dpa fibers, more Maxxa-dominant than TX2094-dominant genes were generally found except for the enhancing category (Fig. 3b, blue points above 50%), whereas the opposite pattern was observed in 10 dpa fibers (red points mostly below 50%). These results do not support a general trend of dominant inheritance, which however, was dependent on developmental stage. Within the inheritance category of transgression, more transgressive up-regulated than down-regulated expressions were consistently found (Fig. 3c and Supplementary Data 3).

### Homoeolog expression changes by regulatory variation

**Fig. 4:**
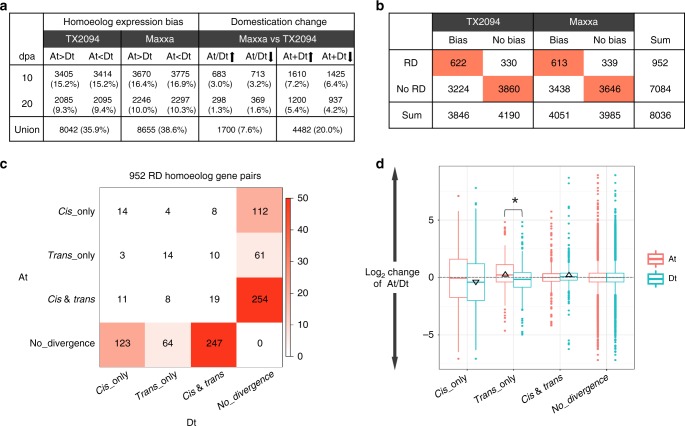
Regulatory evolution and homoeolog expression. **a** The extent of homoeolog expression bias (1st and 2nd columns) and expression changes under domestication (ratios, At/Dt, and total expression, At + Dt; 3rd and 4th columns, respectively) were measured for 22,394 homoeologous gene pairs. **b** Contingency tables between *cis* and *trans* regulatory divergence and homoeolog bias. A pair of homoeologous genes was considered to exhibit regulatory divergence (RD) if at least one homoeolog was found to be a RD gene (1st row). Homoeolog expression bias was characterized each within TX2094 and Maxxa (columns). Cells displaying significant over-representation (Fisher’s exact test; *P* < 0.05) are highlighted. **c** Cross-tabulation of regulatory evolution for 952 RD homoeolog pairs, indicating predominance of *cis* and *trans* effects. Cell color indicates the magnitude of significant over-representation based on −log_10_(*P*-value) of Fisher’s exact test (i.e., *P* = 0.05 is converted to 1.3). **d** Boxplot of homoeolog expression ratio changes under domestication for RD homoeolog pairs. Black triangles indicate significant deviation from zero (Student’s *t*-test; *P* < 0.05), and the asterisk (*) denotes a significantly different ratio between At and Dt homoeologs. Boxplot elements: center line–median; box limits–upper (Q3) and lower (Q1) quartiles; whiskers–smallest and largest non-outlier; points–outliers. The source data underlying Fig. 4d are provided as a Source Data file.

Expression divergence among homoeologous genes, or homoeolog expression bias, has been extensively characterized in plant allopolyploids^35^. To study the extent and variation of homoeolog expression bias (as opposed to ASE of individual homoeologs, discussed up until now) in fibers during domestication, we measured the relative expression of homoeologs (At/Dt) for a total of 22,394 duplicate gene pairs. About one third of these pairs exhibited unequal, or biased, homoeolog expression in either 10 dpa or 20 dpa fibers (8655 pairs in Maxxa and 8042 pairs in TX2094; Fig. 4a, columns of “Homoeolog expression bias”). Consistent with a previous study^30^, more pairs exhibited expression bias at 10 (30–33%) than at 20 dpa (19–20%) in both accessions. Comparing homoeolog ratios between Maxxa and TX2094 identified domestication changes for only 6.2% and 3.0% of gene pairs at 10 and 20 dpa, respectively, suggesting that the relative contribution of homoeologs was mostly conserved under domestication. In addition, the *total* expression for each homoeolog pair (At + Dt) was also compared between Maxxa and TX2094, resulting in 13.4% and 9.5% of gene pairs with significant changes at 10 and 20 dpa, respectively (Fig. 4a, columns of “Domestication change”). Thus, expression changes of individual genes appear to have a larger impact on the *total* expression of homoeologs than their relative ratios.

To evaluate how *cis* and *trans* regulatory variation underlies the dynamics of homoeolog expression patterns, a total of 8036 homoeolog gene pairs were extracted from the 27,816 genes diagnosed with Maxxa/TX2094 SNPs. First, we asked whether the occurrence of regulatory changes under domestication is associated with divergent expression between homoeologous genes. Of 952 homoeolog pairs detected with regulatory changes in at least one homoeolog (i.e., one or both homoeologs belong to the list of 1655 RD genes; see above), about 65% exhibited homoeolog expression bias (613 pairs in Maxxa and 622 pairs in TX2094 fibers; Fig. 4b), significantly higher than the overall percentages of homoeolog bias (50% and 48% out of 8036 pairs, respectively; Fisher’s exact test *P* < 0.05). This suggests that fiber genes that already exhibited homoeolog expression bias in wild cotton were more likely to be modified during domestication. Such regulatory evolution either increased or decreased At/Dt ratios during domestication without significant preference.

Next, we compared regulatory changes in the two co-resident (At and Dt) subgenomes. It has been proposed that allopolyploid cotton *G. hirsutum* has been under asymmetric subgenome selection for improved fiber quality, with more signals of selection detected in the Dt than At subgenome^27^. Under this model, we expected to observe more Dt than At regulatory changes. In contrast, 518 At and 525 Dt genes were observed with regulatory divergence, which are statistically equivalent numbers that counter the earlier study based on genomic scanning for potential selective signals (74 Mb At and 104 Mb Dt region). Here we find that *cis- and trans-* regulatory evolution mostly occurred in only one homoeolog from each pair (Fig. 4c; note that for 861 of 952 pairs, regulatory changes were observed for either but not both of the At and Dt homoeologs). In general, changes in the relative At/Dt ratios and *total* At + Dt homoeolog expression (Supplementary Fig. 3) were unremarkable, except for a small bias toward reduced Maxxa expression when the At homoeologs exhibit no regulatory divergence and the Dt homoeologs exhibit *cis-*only divergence.

Changes in At/Dt ratios were further compared directly between At and Dt regulatory variants (Fig. 4d). *Cis*-only divergence of either homoeolog led to the most pronounced changes in At/Dt ratio (i.e. variability indicated by box size), followed by *trans*-only, and then *cis* + *trans* and no divergence. Significant deviation from zero ratio change was inferred for the *cis*-only effects on Dt (Fig. 4d, black triangle below zero reflecting an increased contribution of Dt versus At homoeolog expression), the *trans*-only effects on At (triangle above zero reflecting an increased expression contribution of At), and the *cis* + *trans* on Dt (above zero). Only the ratio changes associated with *trans*-only effects were significantly different for At and Dt homoeologs (marked by asterisk symbol). These data suggest that the increased expression contribution of At is more associated with *trans*-only regulatory category, while the increased expression contribution of Dt is more associated the *cis*-only regulatory change.

### Elevated sequence divergence with *cis* regulatory divergence

To understand the genetic basis of *cis* divergence, we examined sequence variation and found that RD genes exhibiting *cis*-only variants generally evolve faster with a higher substitution rate in both promoter and coding regions (Supplementary Note 1). As shown in Supplementary Fig. 4, RD genes with *cis* divergence contain more promoter SNPs and indels (within a 2-kb promoter region upstream of the transcription start site) than do genes without *cis* regulatory divergence (*cis*-only or *cis* + *trans* > *trans*-only; Duncan’s multiple range test *P* < 0.05); both *cis*-only and *trans*-only genes tend to display higher substitution rate (dS = 0.0063–0.0070 and dN = 0.0028–0.0037) than those exhibiting *cis* *+* *trans* divergence and non-RD genes (dS = 0.0029–0.0035 and dN = 0.0019–0.0021; Duncan’s multiple range test *P* < 0.05). Between At and Dt homoeologs, comparable amounts of sequence variation were observed except that Dt promoters accumulated more SNPs than did At promoters (6.4 vs. 5.7 SNPs; Student’s *t*-test *P* < 0.05).

### Co-expression network and functional analyses

In biological networks, highly connected genes, or hubs, are thought to have high pleiotropic effects and play essential roles in gene regulation and function. Changes in hub genes are expected to have larger biological impacts than those occurring in genes in the network periphery. To explore the network localization of domestication-related regulatory variations as represented by RD genes, we used the weighted gene co-expression network analysis (WGCNA) to generate the separate wild and domesticated networks from 13 TX2094 and 16 Maxxa fiber RNA-seq datasets (Supplementary Table 1), respectively. Within each network, ranking all genes by either whole network connectivity (k_Total_) or intramodular connectivity (k_Within_), gene set enrichment analysis was applied to determine whether the RD gene sets (*cis*-only, *trans*-only and *cis* + *trans*) were randomly distributed throughout the ranked gene list, or primarily found as network hubs. The TX2094 network exhibited significant enrichment of all RD gene sets (FDR < 0.05) amongst the most highly connected genes, suggesting that regulatory changes under domestication were biased toward targeting genes that were highly connected in wild fiber developmental networks. Likely as a result of these regulatory changes, the network properties of these RD genes were altered to be not enriched as hubs in the Maxxa network. In both networks, the network density (i.e., the connected portion of all possible connections) of RD genes was significantly higher than the same number of randomly selected genes (permutation test *P* < 0.001 by sampling 100 times). As shown in Supplementary Table 4, the subnetwork of *trans*-only genes was consistently denser than the subnetworks of *cis*-only genes (subnetwork density of 0.031 vs. 0.019 in TX2094, and 0.013 vs. 0.008 in Maxxa). One possible explanation is that these *trans* variations may represent genetic or expression changes of a small number of common upstream regulators, so that *trans* affected genes are more functionally associated and interconnected than are the *cis*-only genes, whose *cis* variations are relatively independent. Interestingly, the density of *cis* + *trans* genes is similar to that of *cis*-only genes in TX2094, but in Maxxa it was more similar to the *trans*-only genes (Supplementary Table 4; 0.018 in TX2094 and 0.011 in Maxxa).

**Fig. 5:**
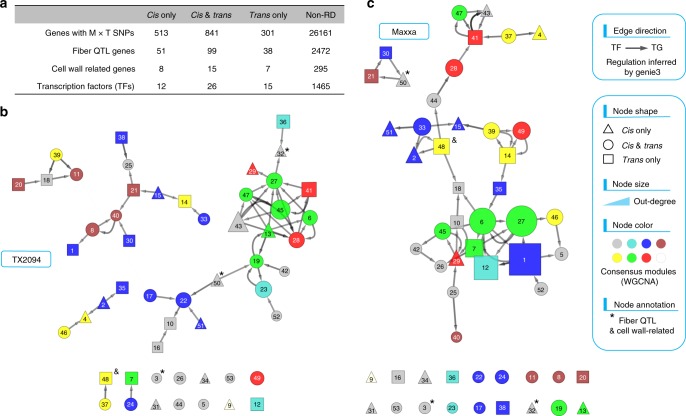
Predicted GRNs of TX2094 and Maxxa fibers. **a** Number of candidate genes within the fiber QTL confidence intervals^28^, from a curated list of cell wall-related genes, and those annotated as TFs. For the total of 53 TFs that belong to the list of 1655 regulatory divergent (RD) genes (see annotation in Supplementary Data 2), a GRN was each inferred for TX2094 (**b**) and Maxxa (**c**) by Genie3 and visualized using Cytoscape. Edge direction represents the regulatory interaction from TF to target TG genes. Node shape represents the categorization of RD patterns. Node size is proportional to number of target genes (i.e., out-degree). Nodes color represents the membership of consensus co-expression gene modules inferred by WGCNA (see Methods and Supplementary Data 2). TFs marked by asterisk (node 3, 32, and 50) and and (48) were found within the confidence intervals of fiber QTLs and related to cell wall synthesis, respectively. The source data underlying Figs. 5b, c are provided as a Source Data file.

Functional enrichment analysis revealed that catalytic activity, DNA metabolic process, and cellular protein localization were the most enriched gene ontology (GO) classifications for fiber expressed genes (the union of 55,551 with read depth above 5 at either stage among 66,610 genes; Supplementary Data 4; adjusted *P* *<* 0.05). For the differentially expressed genes between Maxxa and TX2094 fibers, catalytic and metabolic activities such as oxidoreductase and steroid dehydrogenase activity were enriched at both developmental stages of 10 and 20 dpa, whereas cytoskeleton-related molecular function and biological processes were specifically enriched in 20 dpa fibers (Supplementary Data 5; adjusted *P* *<* 0.05). Against the reference list of 27,816 genes containing diagnostic SNPs between Maxxa and TX2094 alleles (Supplementary Data 6; adjusted *P* *<* 0.05), genes exhibiting regulatory divergence under domestication (1655 RD genes) are mainly associated with heme binding, oxidoreductase and transferase activity, as well as the cellular components of cytoskeleton, cell wall, and clathrin coat. Among these, 188 RD genes were identified as candidate fiber domestication genes from a recent QTL study of Maxxa versus TX2094^28^, representing a significant overlap between these two gene lists (Fisher’s exact test *P* < 0.05; Fig. 5a and Supplementary Data 2 column of “fiber QTL genes”). With respect to different categories of regulatory divergence, *cis*-only genes were specifically enriched for oxidoreductase activity and clathrin-coated vesicle, *trans*-only genes were specifically enriched for xyloglucan:xyloglucosyl transferase activity, lipid biosynthetic process, and cellular components including cell wall, cytoskeleton, microtubule and kinesin complex, and the *cis* + *trans* genes are mainly involved in the regulation of signal transduction and response to stimuli (Supplementary Fig. 5 and Supplementary Data 6; adjusted *P* *<* 0.05).

### Exploiting gene regulatory networks for functional discovery

To further explore the potential for functional discovery during fiber domestication, we constructed gene regulatory networks (GRNs) of TX2094 and Maxxa using Genie3^36^, which predicts directed regulatory links from transcription factors (TFs) to their target genes (TGs). Among a total of 1518 TFs included for GRN construction, 53 were detected with regulatory variation, including 12 *cis*-only, 26 *cis* + *trans*, and 15 *trans*-only RD genes (Fig. 5a and Supplementary Data 2). Because more TGs were predicted for these RD TFs than the non-RD TFs in both fiber networks (Wilcoxon test *P* < 0.05), it is reasonable to speculate that RD TFs act as causal transcriptional regulators involved in fiber domestication. As visualized by Cytoscape^37^, the TX2094 subnetwork of these 53 RD TFs is composed of four disconnected clusters of genes (Fig. 5b), whereas the majority of Maxxa nodes were connected into one large cluster in the network (Fig. 5c). This increased level of interconnectivity in domesticated vs. wild cotton is consistent with the previous report in seed development^31^, as well as a separate study of fiber co-expression networks (unpublished). Functional implications of specific TFs and their network properties were detailed in Supplementary Note 2.

## Discussion

The domestication and improvement of cotton fiber concerns a structure that is only a single cell, yet one that has an extraordinarily complex genetic architecture, as evidenced by multiple sources of developmental, genetic, and comparative evidence^4,26–28,30–32,38^. Here we shed light on the regulatory dimension of this complexity by demonstrating that 15% of the genes in the fiber transcriptome have experienced some form of regulatory alteration between a pair of wild and domesticated *G. hirsutum* accessions (TX2094 and Maxxa, respectively). Using their reciprocal hybrids, we provide a systematic catalog of *cis* and *trans* regulatory variants that underlie the extraordinary transformation in the single-celled phenotype (Fig. 1) resulting from cotton domestication.

Our study shows that *cis* and *trans* effects account for 36–48% and 52–64% of regulatory divergence accompanying fiber domestication, respectively (Supplementary Data 1), percentages that are remarkably similar to those reported (~45% vs. ~55%) for maize vs. teosinte^16^. These observations suggest almost equally important roles of these two regulatory mechanisms in the evolutionary process at this temporal scale of divergence. In maize, however, nearly 80% of large expression divergence (|***A***| > 5) was found due to *cis* contributions^16^, and the selection candidate genes cataloged^39^ were much more significantly enriched in *cis* than *trans* affected genes. In cotton, although we detected more *cis*-only than *trans*-only genes (1.2–2 fold), *cis* contributions to large parental expression differences between TX2094 and Maxxa account for only 42–53% of the total regulatory changes implicated in domestication (Fig. 2b). Moreover, *trans* effects were more highly correlated with expression divergence than were *cis* effects, in line with the significant role of *trans* contribution as has been reported for intraspecific expression divergence (see below). Interestingly, 53% of the genes that are differentially expressed between these wild and domesticated cotton accessions show higher expression in the domesticated, whereas in their F_1_ hybrids only ~30% of genes exhibit this same bias (Supplementary Table 3). This result underscores the importance of both *cis* and *trans* effects contributing to expression divergence, with more TX2094 > Maxxa allelic expressions by *cis* in F_1_s and the opposite pattern by *trans*. The antagonistic action of *cis* and *trans* effects is also evidenced by their higher frequency than that of reinforcing changes (Fig. 1a). Although this observation and similar reports elsewhere^40–43^ may partly reflect an intrinsic bias of the standard ASE method, as recently noted^33^, the negative correlation between *cis* and *trans* effects remained significant when we applied a cross-replicate method to eliminate the bias. Although the prevalence of negative correlation awaits a systematic re-evaluation of previous reports using the cross-replicate approach or other methods not subjected to bias (such as eQTL mapping)^33^, many mechanisms underlying the antagonistic effects have been proposed^34^, including stabilizing selection for compensatory changes^40,43,44^, buffering from various forms of gene network feedbacks (e.g. negative feedback at mRNA level^45^ and at translation level^46,47^), and communication between chromosomes (termed transvection in animals)^48^.

Previous studies in yeast^41,42,49^ and *Drosophila*^13,50^ have shown that the proportion of *cis* and *trans* regulatory variation usually correlates with the scale of phylogenetic divergence, such that the relative contribution of *cis* effects increases with time, with the corollary that *trans* effects are mostly observed between different populations of the same species. Reasons for this correlation are not entirely clear, but may be related to strong selection during speciation on *cis* variation, the multiple pleiotropic effects of *trans* mutations, and the different modes of selection on linked vs. dispersed regulatory variants^34^. In plants, this correlation is also supported by numerous interspecific^18,51,52^ and intraspecific studies^14,15,53^, but exceptions are also common^17,20,54^.

Our results here are congruent with the expectation that *trans*-regulatory differences should have a greater impact within species (here, *G. hirsutum*); however, the question remains as to how *trans* variations have quickly accumulated between these two relatively recently diverged (circa 5000–8000 years ago) accessions, in contrast to evidence from other systems such as maize^54,55^. We suggest that this likely reflects the allopolyploid nature of the cotton genome. That is, the genome of *G. hirsutum* is composed of two suites of genes that diverged 5–10 million years before being reunited in a common nucleus 1–2 million years ago^22^. Notably, and somewhat uncommonly, the two genomes that comprise the allopolyploid are two-fold different in size but have about the same genic content. The result is a mostly duplicated syntenic genome whose genic complement is largely intact between subgenomes but whose local genic environment may be different due to the vastly different complements of TEs and other repetitive sequences. It is worth noting that different parental TE loads and their relative distribution between subgenomes have been the most popular explanation for biased homoeolog expression and biased genome fractionation^56–58^, which likely further complicates regulatory circuits in allopolyploids. Consequently, with the onset of polyploidy, *trans-*regulated genes instantaneously acquired highly similar, yet still divergent, *trans* factors capable of interacting with both homoeologous suites of *cis* elements. We suggest that this facet of allopolyploidy vastly increased the opportunity for novel *cis*-*trans* interactions due to overlapping, but divergent, regulatory programs and TF binding sites^24^, ultimately creating novel evolutionary possibilities, especially for *trans* variants. This phenomenon of enhanced *trans* regulatory evolution may be a general and previously unrecognized feature of polyploidy, perhaps helping to explain evolutionary novelty in recently formed allopolyploid plants.

The domestication changes we observed for both the aggregate homoeolog expression and the relative expression among homoeologs suggest that the allopolyploid genome amplifies the combinatorial complexity of regulatory evolution. One puzzling observation is that, while biased homoeolog expression in wild cotton was enriched in all forms of regulatory change (*cis*-only, *trans*-only, *cis* + *trans*), this general enrichment was vastly reduced in the domesticated accession. Neither the genesis nor the functional implications of this reduction are clear, although we speculate that it may be related to the observation that cotton domestication appears to lead to increased interconnectivity between genes and homoeologs^31^, suggestive of tighter gene expression coordination. In the context of cotton fiber gene co-expression networks, by contrasting the regulatory variation between homoeologous genes, we asked whether, and to what extent, the A- and D- subgenomes were independently regulated under domestication, in terms of the mode and scale of *cis-trans* interactions. Our major observations (Fig. 4) led to the conclusion that the subgenomes of allotetraploid cotton are to some extent uncoupled at the transcriptional level, notwithstanding the increased opportunity for *trans* regulatory evolution discussed in the previous section.

One key outcome of our study is a catalog of fiber-expressed genes with *cis* and *trans* regulatory variants that have been altered through divergence and domestication (1655 RD genes). These genes are enriched in QTL regions responsible for important fiber characteristics (i.e., length, quality and color) that differentiate Maxxa and TX2094^28^, suggesting a significant role of regulatory evolution in fiber phenotypic evolution. Although other studies have highlighted the selective relevance of *cis* variation^2,11^, no obvious difference was found among the three RD categories (*cis*-only, *trans*-only, *cis* + *trans*) in their association with candidate selection genes, transcription factors and cell wall-related genes (Fig. 5a). However, compared to *cis* affected RD genes that were mainly involved in signal transduction and redox homoeostasis, rather interestingly, the *trans*-only genes were specifically enriched for key categories of constitutive fiber enzymatic and structural genes, including xyloglucan:xyloglucosyl transferase activity, lipid biosynthetic process, and cellular components of cell wall, cytoskeleton, microtubule and kinesin complex. Likely responsive to a small number of pleiotropic *trans* mutations, these *trans*-only genes were also found to display a higher level of network connection and functional association than were *cis*-affected genes (Supplementary Table 4). Considering their central functions in cotton fiber biology and prospects for molecular genetic-informed breeding, it is crucial to recognize the *trans* responsiveness of genes in modifying fiber phenotypes. Although pinpointing upstream regulators (transcription factors, sRNA, chromatin remodelers, etc.) and causative mutations remains challenging, we anticipate that that this will soon be facilitated by emerging technologies, such as DNase-seq and Hi–C^27,38^. Here we explored potential regulatory links among the transcription factors that exhibit *cis* and/or *trans* variation, as well as the global characteristics in gene network topology. The resulting regulatory networks together with the catalog of RD genes provide a useful resource for further work aimed at a deeper understanding of cotton fiber biology and evolution, and may also have implications for breeding and crop improvement.

## Methods

### Plant materials

Four accessions of *Gossypium hirsutum* were used in this study, including a wild accession, TX2094, an elite cultivar, Acala Maxxa (hereafter Maxxa), and their reciprocal F_1_ hybrids Maxxa × TX2094 (M × T) and TX2094 × Maxxa (T × M). Three to four replicates of each accession were grown in the Bessey Hall Greenhouse at Iowa State University (Ames, Iowa, USA). At 10 and 20 days post-anthesis (dpa), ovules were collected, and immediately frozen in liquid nitrogen and stored in −80 °C. These two developmental stages represent the midpoint of the duration of primary cell elongation and the transition period to secondary wall synthesis, respectively.

### RNA extraction and sequencing

Cotton fibers were isolated from ovules using a liquid nitrogen/glass bead shearing approach^59^. Subsequent total RNA extractions were performed using Sigma spectrum plant total RNA kit (Cat No. STRN50), and quantified on a BioAnalyzer (Agilent, Palo Alto, CA). mRNA libraries were prepared using the Illumina TruSeq RNA Library Prep Kit (Illumina, San Diego, CA, USA) and sequenced on three Hiseq 2500 lanes with paired-end 125-cycle sequencing. A total of 27 libraries with a minimum of 18 million reads per sample were generated (Supplementary Table 1).

### Processing of RNA-seq datasets

Trim Galore (https://www.bioinformatics.babraham.ac.uk/projects/trim_galore/), a wrapper script of Cutadapt^60^ and FastQC (https://www.bioinformatics.babraham.ac.uk/projects/fastqc/) was used to filter and trim raw RNA-seq data. Filtered RNA-seq reads were processed by HyLiTE^61^ to produce tables of parental and allelic expression data in a single step. First, all libraries of RNA-seq reads were mapped against the reference *G. hirsutum* genome^62^ using bowtie2^63^ with option –N 1. Next, using the*.pileup* file generated by samtools^64^, SNPs that diagnostic of the two parents were detected and used to determine the parental origin of hybrid reads. The final output files of HyLiTE contained the read count tables of total and allelic gene expression in the hybrids and parental accessions. The percentages of F_1_ allelic reads assigned to Maxxa and TX2094 are summarized in Supplementary Table 2.

### Differential gene expression analysis

Analysis of differential gene expression was conducted in R (R Foundation for Statistical Computing, Vienna, Austria) with the package DESeq2^65^. Pairwise comparisons of gene expression were assessed between accessions at the same fiber developmental stage, and between Maxxa and TX2094 alleles in the F_1_ hybrids (Supplementary Table 3). For each pair of A- and D-genome homoeolog genes (see detection method below), homoeolog expression ratios were calculated to infer homoeolog expression bias (i.e. unequal homoeologous expression) with DESeq2, and directly compared between Maxxa and TX2094 using Student’s *t* test. The distribution of *p*-values was controlled for a false discovery rate (*q*-value) by the Benjamini-Hochberg method^66^ at *α* = 0.05.

### Characterization of *cis* and *trans* regulatory divergence

The combination of parental gene expression and F_1_ allelic expression data was used to characterize *cis* and *trans* effects (Fig. 1)^18,43^. First, the overall contributions of *cis* and *trans* variants were measured by log_2_ ratios of the parental divergence between Maxxa and TX2094 (***A*** = log_2_(M/T)), and *cis* effects were measured by log_2_ ratios of the corresponding allelic divergence in F_1_ hybrids (***B*** = log_2_(m/t)); *trans* effects thereby can be derived by subtracting the allelic divergence from the parental divergence (***A***-***B***). Although this standard method has been widely used since^13^, it was recently noted that estimating *cis* and *trans* effects from the same measures of F_1_ allelic divergence is intrinsically biased to introduce an artifactual negative correlation between them, whereas a cross-replicate approach can eliminate the bias^33^. For the standard method, allelic measures from all three biological replicates of M × T or T × M were used to calculate ***B*** and subsequently ***A***-***B***. For the cross-replicate approach, one biological replicate was used for estimating ***B***, while a different replicate was used for estimating ***B’*** and then ***A***-***B***’; the *cis* and *trans* effects were thus independently measured with random errors to each other. All six possible cross-replicate combinations of three replicates were analyzed.

Next, we classified gene into seven categories of regulatory evolution based on the statistical significance of ***A***, ***B***, and ***A*** vs. *B*^18,43^, as follows and illustrated in Fig. 1. Significant expression divergence (***A*** ≠ 0) and *cis* effects (***B*** ≠ 0) were determined using DESeq2^65^, and the presence of *trans* effects (***A*** ≠ ***B***) was tested using Student’s t-tests; both followed by multiple testing correction using the Benjamini-Hochberg procedure^66^. Because replicates are needed to measure variation and the actual values of ***A***-***B*** is not involved, the standard inference of ***B*** was applied for categorization. If a gene exhibited allelic divergence in the F1 equivalent to that between Maxxa and TX2094, the regulatory divergence of this gene was considered to be caused by only *cis* effects (category I: ***A*** **=** ***B***, ***A*** ≠ 0, ***B*** ≠ 0). If equal allelic expression was observed in F1 in spite of significant divergence between parents, a gene was considered having only *trans* effects (II: ***A*** ≠ ***B***, ***A*** ≠ 0, ***B*** = 0). When parental expression divergence could not be attributed to only *cis* or only *trans* effects (***A*** ≠ ***B***, ***A*** ≠ 0, ***B*** ≠ 0), the directions of *cis* and *trans* effects were compared to identify enhancing (category III) and compensating (category IV) interactions, depending on whether the *cis* and *trans* effects on a gene were in the same or opposite directions, respectively. When equal expression was found between the parents but not in the F1, *cis* and *trans* variants were considered fully compensatory (category V; ***A*** ≠ ***B***, ***A*** ***=*** 0, ***B*** ≠ 0). Without expression divergence detected anywhere, conserved expression was defined (category VI; ***A*** = ***B***, ***A*** = 0, ***B*** = 0). The remaining expression patterns statistically conflict with each other, and hence are classified as ambiguous (category VII). A series of different log_2_ fold-change cutoffs were tested to evaluate the consistency of categorical patterns.

If the regulatory pattern of categories I–V was categorized consistently between reciprocal F1 hybrids (M × T and T × M) for a gene at either developmental stage, this gene was considered to display regulatory divergence (RD) during fiber domestication. This list of RD genes was further categorized to display *cis* only, *trans* only or both (*cis* and *trans*) types of regulatory divergence, based upon categorization results from two developmental stages. That is, when a RD gene was characterized only by category I (at both 10 and 20 dpa, or at either stage with no divergence inferred at the other stage), this case was considered to be *cis* only; when a RD gene was characterized only by category II, the case was considered to be *trans* only; all other cases were considered be *cis* and *trans* RD genes.

### Gene coding region and promoter sequence divergence

Coding sequence SNPs between the two parents, Maxxa and TX2094, were identified by HyLiTE from the RNA-seq datasets as described above. These SNPs were used to replace the TM-1^62^ reference bases at variable sites to generate Maxxa and TX2094 orthologous sequences, and further classified as synonymous or nonsynonymous SNPs using custom R scripts (https://github.com/Wendellab/CisTransRegulation). Evolutionary rates for both nonsynonymous (dN) and synonymous (dS) sites were calculated using the codeml program implemented in the PAML package^67^ using runmode = −2.

To compare promoter sequences, we downloaded the genomic DNA data from the NCBI Sequence Read Archive (SRA; http://www.ncbi.nlm.nih.gov/sra) for Maxxa (SRA accession number SRR617482) and TX2094 (SRR3560138-3560140). After quality filtering and trimming using SOAPnuke v1.5.2^68^, clean reads were mapped to the reference genome of the cultivated Upland cotton TM-1^62^ using BWA v0.7.10^69^ with default parameters. Reads around indels from the BWA alignment were realigned with the tools of RealignerTargetCreator and IndelRealigner in the Genome Analysis Toolkit (GATK)^70^. SNP and indel calling was performed with GATK and FreeBayes^71^. To ensure high-quality SNPs and indels, only variation detected by both software tools with a sequencing depth of at least 10 was retained for further analysis. Using the snpEff software^72^, promoter SNPs and indels within a 2-kb region upstream of the transcription start site were annotated, and only homozygous variants were kept to calculate sequence divergence in terms of the number of SNPs or indels per Kb.

### Genome-wide identification of homoeologous gene pairs

Given the repeated paleopolyploidy events in the evolutionary history of *Gossypium* genomes^73^, we applied a high-confidence syntenic approach to infer orthologs in the diploid *G. raimondii* and the two subgenomes of *G. hirsutum* (treated separately). First, three sets of proteins were subjected to orthologous gene group interference using OrthoFinder v.2.1.2^74^ with the default program settings. Orthologous groups consisting of one gene from each set were then treated as known homologs as input for MCScanX_h^75^ to score syntenic homologs and output collinear blocks. Next, the syntenic homologs were connected into clusters using custom python scripts (https://github.com/Wendellab/CisTransRegulation/tree/master/analysis/Collinear_Orthologs), where nodes are genes and edges indicate the collinear relationships. The size of a cluster depends on the number of related collinear blocks, and only clusters containing one gene (node) per (sub)genome were retrained as high-confidence multi-genome anchors. This step was designed to eliminate collinear blocks originating from paleopolyploidy events. Finally, we used MCScanX to identify all inter-species collinear blocks (-b 2) with at least three genes in each collinear block (-s 3). The final output was further restricted to those blocks containing at least one pair of genes belonging to the anchors. This process created curated gene triplets each containing one gene from the diploid *G. raimondii* and one from each of the two subgenomes of *G. hirsutum*.

### Co-expression and regulatory gene network analysis

We downloaded additional RNA-seq data for developing fibers of Maxxa and TX2094 from NCBI Short Read Archive (see accession numbers in Supplementary Table 1). Together with the samples generated in this study, a total of 13 TX2094 and 16 Maxxa fiber RNA-seq datasets were subjected to gene network construction. Raw reads were preprocessed and mapped against the reference *G. hirsutum* genome^62^ as described above. The resulting read count tables were normalized by *rlog* transformation, built in DESeq2^65^. The WGCNA package in R^76^ was used to build individual weighted co-expression networks for Maxxa, TX2094 and their consensus network with an optimized power $\beta$ = 20. The whole network connectivity (k_Total_) and intramodular connectivity (k_Within_) were calculated with WGCNA function *signedKME*. To assess whether RD genes are enriched at the top of the ranked list of connectivity, gene set enrichment analysis was applied using the R package clusterProfiler^77^.

Of the 27,816 genes detected with parent-diagnostic SNPs, 1518 transcription factors (TFs) and corresponding families were annotated by PlantTFDB v4.0^78^. Given the normalized expression profiles and the list of TFs, putative regulatory links from TFs to all other genes were estimated using Genie3^36^ with default parameters, and only regulatory links with a prediction weight above 0.005 were retained for constructing a gene regulatory network (GRN). For each node (gene) in the network, in- and out-degree represented the number of edges (regulatory links) directed to and from this node, respectively. For each of the 52 TFs that overlap with the list of RD genes, GO enrichment analysis of its target genes was performed using the R package clusterProfiler^77^ with the following parameters: pvalueCutoff = 0.05, pAdjustMethod = BH, minGSSize = 10, maxGSSize = 500, qvalueCutoff = 0.05. The top three GO terms for each TF are summarized in Supplementary Data 2.

### Reporting summary

Further information on research design is available in the Nature Research Reporting Summary linked to this article.

## Supplementary information

Supplementary Information

Peer Review File

Reporting Summary

Description of Additional Supplementary Files

Supplementary Dataset 1

Supplementary Dataset 2

Supplementary Dataset 3

Supplementary Dataset 4

Supplementary Dataset 5

Supplementary Dataset 6

## Data Availability

Data supporting the findings of this work are available within the paper and its Supplementary Information files. A reporting summary for this Article is available as a Supplementary Information file. The datasets generated and analyzed during the current study are available from the corresponding author upon request. Raw RNA-seq data generated during this study have been deposited in the NCBI Short Read Archive under PRJNA529497 (https://www.ncbi.nlm.nih.gov/bioproject/PRJNA529497). The source data underlying Figs. 1b, 2, 3, 4d, 5b, and 5c, as well as Supplementary Figs. 2, 4 are provided as a Source Data file.

## Source Data

Source Data

## Code availability

Custom R scripts are available at https://github.com/Wendellab/CisTransRegulation.
