# Supplementary material for: Unraveling cis and trans regulatory evolution during cotton domestication
Source: Nat Commun. 2019 Nov 27;10:5399. doi: 10.1038/s41467-019-13386-w (PMC6881400; doi:10.1038/s41467-019-13386-w)
Supplement: Supplementary file 1 — Supplementary Information [file 41467_2019_13386_MOESM1_ESM.pdf]

# **Unraveling *cis* and *trans* regulatory evolution during cotton domestication**

Bao *et al.*

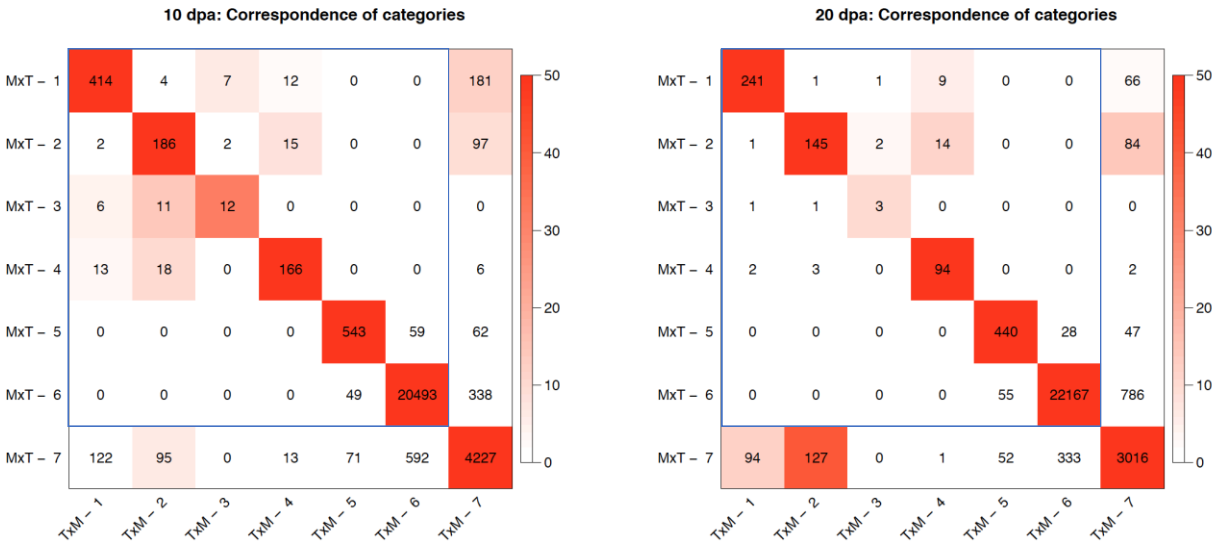


**Supplementary Figure 1. Correspondence of *cis* and *trans* regulatory categorization between M×T and T×M.** Cross-tabulation of gene numbers is shown at 10 and 20 dpa. Pearson's Chi-square test of independence revealed significant association between M×T and T×M for both contingency tables ($P < 0.05$). Based on Fisher's exact test, statistical significance of enrichment is shown by the color of each cell. Darkest red cells indicate the most significantly enriched overlap between their corresponding row (M×T) and column (T×M) categories. Inside the boxes excluding ambiguous genes, over 99% of genes are located in the diagonal cells.

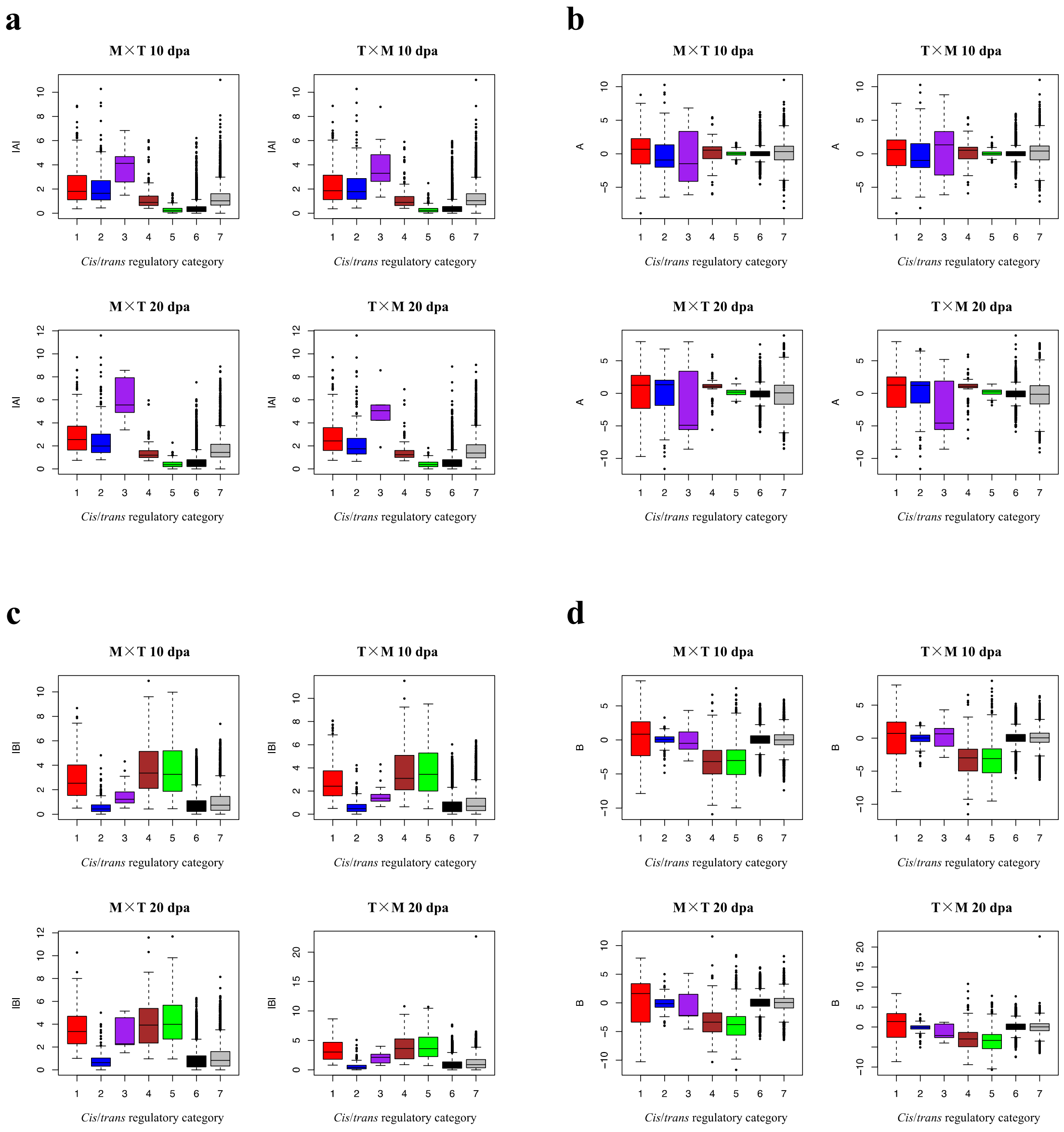


**Supplementary Figure 2. Boxplots of seven *cis* and *trans* regulatory categories corresponding to parental expression divergence and *cis* regulatory divergence.** As in Figure 2c-f, the magnitude and direction of parental expression divergence (**a** - |***A***| and **b** - ***A***) and *cis* regulatory divergence (**c** - |***B***| and **d** - ***B***) were plotted for four $F_1$ hybrid samples within each panel. M×T, Maxxa × TX2094; T×M, TX2094 × Maxxa. Boxplot elements: center line - median; box limits - upper (Q3) and lower (Q1) quartiles; whiskers - smallest and largest non-outlier; points - outliers. Source data are provided as a Source Data file.

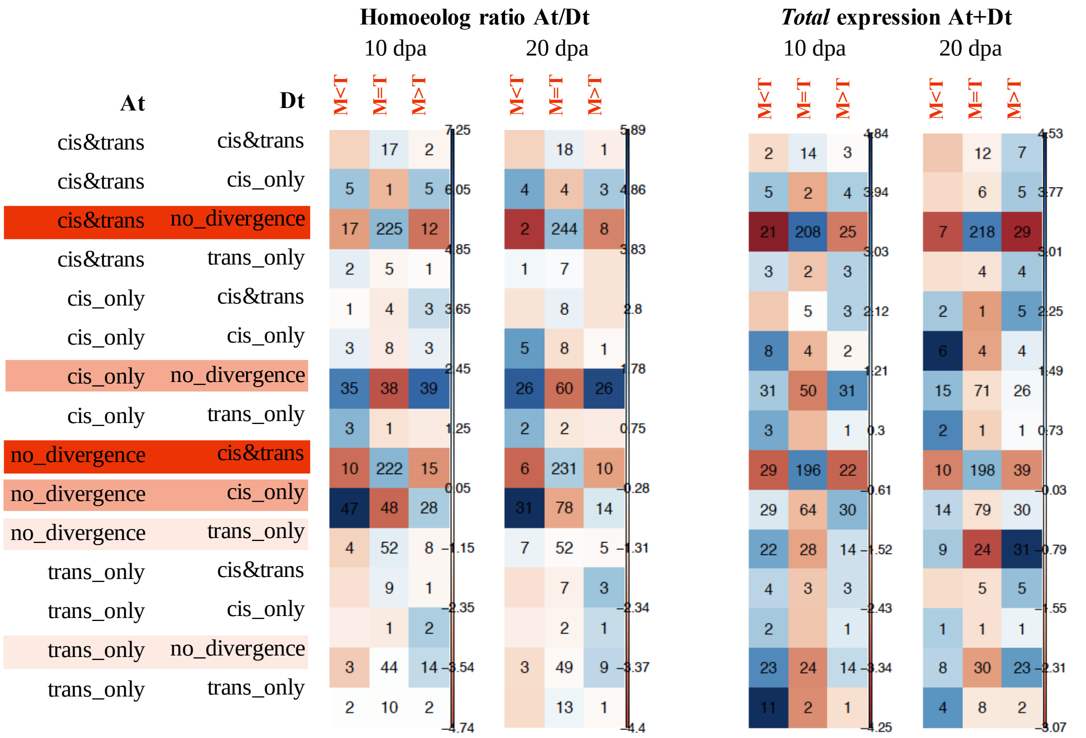


**Supplementary Figure 3. Homoeolog expression patterns altered by At and Dt regulatory variation.** On the left, 15 combinatorial patterns of At and Dt regulatory variations are listed, with the most overrepresented patterns as colored in Figure 4c. Corresponding to each combinatorial pattern, expression changes between Maxxa and TX2094 in At/Dt ratio and At+Dt *total* expression are tabulated at 10 and 20 dpa. The color of each cell shows the magnitude of gene enrichment (blue) and depletion (red) based on residuals of Pearson's Chi-square test of independence (blue indicates a positive residual, i.e., more genes observed than expected, and red indicates fewer genes than expected).

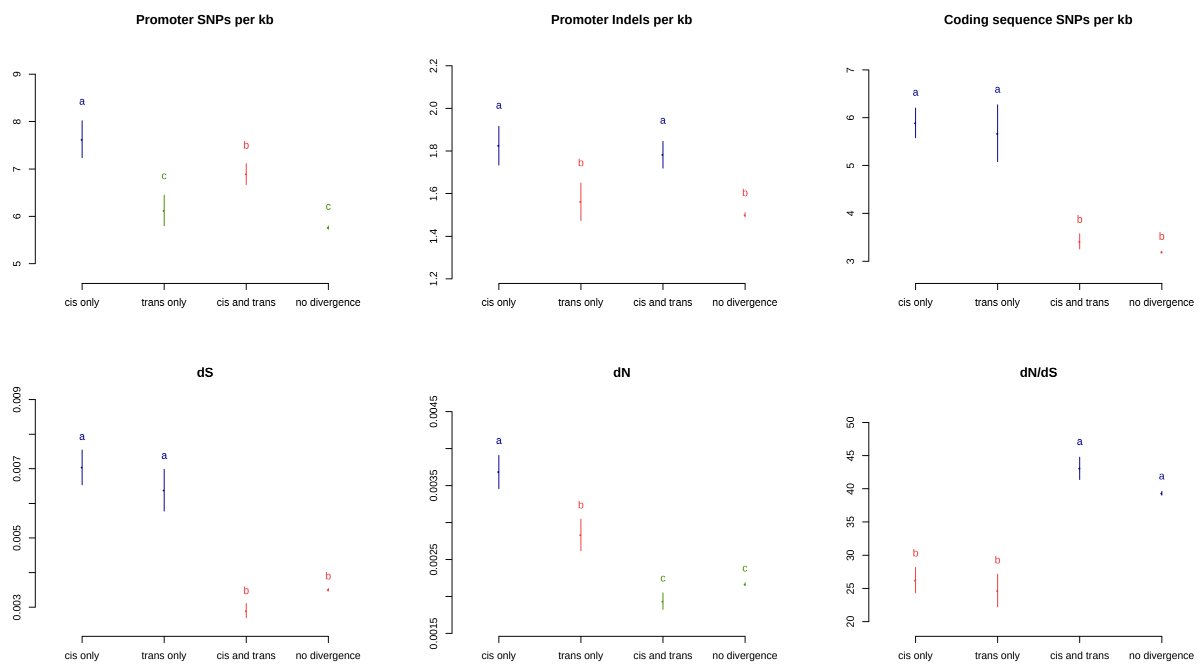


**Supplementary Figure 4. Relationships between sequence divergence and regulatory divergence.** Measures of DNA sequence divergence, including promoter SNPs and indels, coding region SNPs, synonymous rate (*dS*), nonsynonymous rate (*dN*) and *dN*/*dS*, were contrasted between genes of different regulatory patterns using Duncan’s multiple range test, with error bars depicting standard errors. The different letters (e.g., a, b, c) denote significant differences ($P < 0.05$). Source data are provided as a Source Data file.

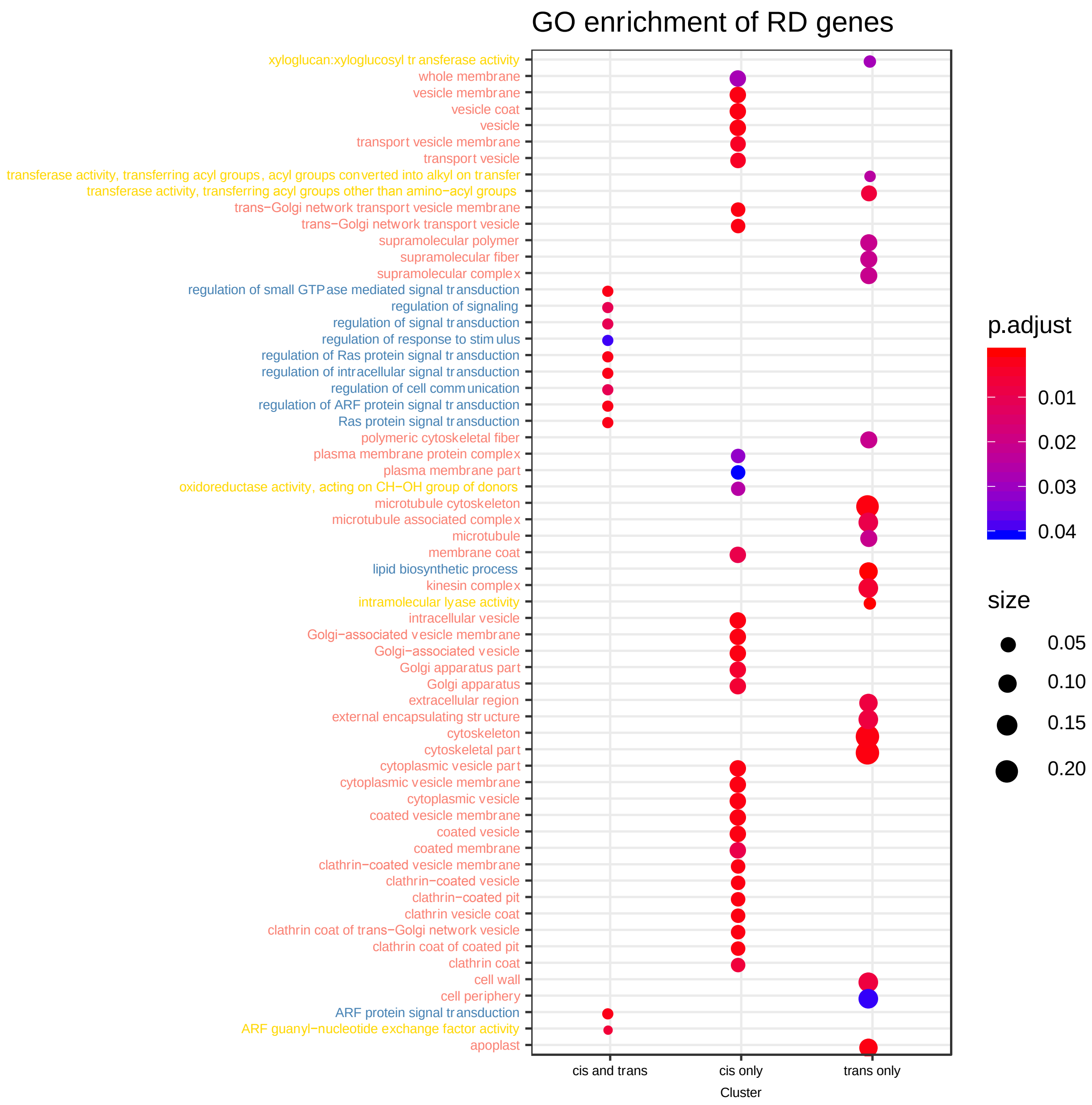


**Supplementary Figure 5. GO enrichment of regulatory divergent genes.** Enriched GO terms are compared for three different categories of regulatory divergent (RD) genes on the x-axis, and the corresponding ontology of molecular function (MF - gold), biological process (BP – steel blue), and cellular component (CC - salmon) are colored on the y-axis. Dot size and dot color represent gene ratio and adjusted p-value, respectively.

## Supplementary Table 1. Summary of fiber RNA-seq samples included in this study.

| SRA | accession | dpa | rep | Total reads number | Filtered/mapped reads number | % | note |
|---|---|---|---|---|---|---|---|
| **Datasets generated in this work** | | | | | | | |
| SRR8797010 | M×T | 10 | 1 | 10,756,588 | 6,570,501 | 61.08% | |
| SRR8797009 | M×T | 20 | 1 | 8,985,380 | 6,105,101 | 67.94% | |
| SRR8797008 | M×T | 10 | 2 | 10,110,567 | 6,009,681 | 59.44% | |
| SRR8797007 | M×T | 20 | 2 | 11,494,379 | 6,918,189 | 60.19% | |
| SRR8797006 | M×T | 10 | 3 | 10,740,520 | 6,061,867 | 56.44% | |
| SRR8797005 | M×T | 20 | 3 | 10,778,185 | 6,747,724 | 62.61% | |
| SRR8797004 | T×M | 10 | 1 | 10,026,565 | 5,977,375 | 59.62% | |
| SRR8797003 | T×M | 20 | 1 | 9,685,175 | 5,926,936 | 61.20% | |
| SRR8797012 | T×M | 10 | 2 | 10,000,290 | 5,774,898 | 57.75% | |
| SRR8797011 | T×M | 20 | 2 | 10,178,768 | 6,186,586 | 60.78% | |
| SRR8797028 | T×M | 10 | 3 | 10,815,961 | 6,520,713 | 60.29% | |
| SRR8797027 | T×M | 20 | 3 | 10,780,275 | 6,519,842 | 60.48% | |
| SRR8797030 | Maxxa | 10 | 1 | 9,839,562 | 5,491,071 | 55.81% | |
| SRR8797029 | Maxxa | 20 | 1 | 9,986,304 | 5,731,427 | 57.39% | |
| SRR8797024 | Maxxa | 10 | 2 | 11,374,202 | 5,972,051 | 52.51% | |
| SRR8797023 | Maxxa | 20 | 2 | 10,779,389 | 5,977,830 | 55.46% | & |
| SRR8797026 | Maxxa | 10 | 3 | 10,593,243 | 5,845,661 | 55.18% | |
| SRR8797025 | Maxxa | 20 | 3 | 10,045,980 | 6,351,115 | 63.22% | |
| SRR8797022 | Maxxa | 10 | 4 | 10,773,224 | 6,202,329 | 57.57% | |
| SRR8797021 | Maxxa | 20 | 4 | 9,646,587 | 5,389,200 | 55.87% | |
| SRR8797013 | TX2094 | 10 | 1 | 11,309,911 | 6,303,933 | 55.74% | |
| SRR8797014 | TX2094 | 20 | 1 | 10,770,070 | 4,334,720 | 40.25% | * |
| SRR8797015 | TX2094 | 10 | 2 | 9,971,759 | 6,100,029 | 61.17% | |
| SRR8797016 | TX2094 | 20 | 2 | 11,483,824 | 3,929,115 | 34.21% | *& |
| SRR8797017 | TX2094 | 10 | 3 | 11,206,757 | 5,701,339 | 50.87% | |
| SRR8797018 | TX2094 | 20 | 3 | 10,740,479 | 4,136,004 | 38.51% | * |
| SRR8797019 | TX2094 | 20 | 4 | 27,700,290 | 14,260,153 | 51.48% | |
| **Downloaded datasets** | | | | | | | |
| SRR8797020 | TX2094 | 10 | JJ | 21,052,971 | 12,383,164 | 58.82% | |
| SRR203242 | Maxxa | 10 | BYU | 13,555,520 | 10,240,026 | 75.54% | |
| SRR203244 | TX2094 | 10 | BYU | 12,954,154 | 8,960,954 | 69.17% | |
| SRR203247 | Maxxa | 20 | BYU | 11,862,948 | 7,340,486 | 61.88% | |
| SRR611435 | TX2094 | 20 | MJY | 23,925,569 | 10,409,586 | 43.51% | |
| SRR611436 | Maxxa | 10 | MJY | 20,872,445 | 10,005,262 | 47.94% | |
| SRR611443 | Maxxa | 20 | MJY | 19,740,447 | 5,714,567 | 28.95% | |
| SRR611445 | TX2094 | 10 | MJY | 16,282,320 | 8,718,768 | 53.55% | |
| SRR8837952 | Maxxa | 5 | SRB | 44,718,091 | 32,057,851 | 71.69% | |
| SRR8837953 | Maxxa | 10 | SRB | 29,367,962 | 19,629,588 | 66.84% | |
| SRR8837954 | Maxxa | 15 | SRB | 31,686,165 | 14,551,393 | 45.92% | |
| SRR8837955 | Maxxa | 20 | SRB | 2,769,552 | 1,622,905 | 58.60% | |
| SRR8837969 | TX2094 | 5 | SRB | 27,776,650 | 15,530,616 | 55.91% | |
| SRR8837971 | TX2094 | 15 | SRB | 26,232,665 | 18,863,550 | 71.91% | |
| SRR8837970 | TX2094 | 20 | SRB | 38162310 | 4,768,072 | 12.49% | * |

* Lower mapping rates due to higher sequence duplication levels, indicating some kind of enrichment bias in generating RNA-seq libraries for TX2094 20dpa samples.
& Included for SNP detection but excluded from other analyses (including DE, cis/trans and network analysis) due to incorrect labeling, as both 20 dpa samples were clustered with corresponding 10 dpa samples.

**Supplementary Table 2. Summary of $F_1$ allelic read assignment.**

| | Maxxa | | TX2094 | | Unassigned | | |
|---|---|---|---|---|---|---|---|
| Sample | number | % | number | % | number | % | Total |
| M×T.10dpa.1 | 965,758 | 7.3% | 1,011,273 | 7.7% | 11,163,977 | 85.0% | 13,141,008 |
| M×T.10dpa.2 | 885,338 | 7.4% | 927,945 | 7.7% | 10,206,087 | 84.9% | 12,019,370 |
| M×T.10dpa.3 | 901,602 | 7.4% | 931,438 | 7.7% | 10,290,728 | 84.9% | 12,123,768 |
| T×M.10dpa.1 | 871,128 | 7.3% | 904,285 | 7.6% | 10,179,339 | 85.1% | 11,954,752 |
| T×M.10dpa.2 | 851,544 | 7.4% | 878,906 | 7.6% | 9,819,356 | 85.0% | 11,549,806 |
| T×M.10dpa.3 | 959,844 | 7.4% | 998,359 | 7.7% | 11,083,233 | 85.0% | 13,041,436 |
| M×T.20dpa.1 | 983,266 | 8.1% | 966,027 | 7.9% | 10,260,917 | 84.0% | 12,210,210 |
| M×T.20dpa.2 | 1,037,788 | 7.5% | 1,069,653 | 7.7% | 11,728,953 | 84.8% | 13,836,394 |
| M×T.20dpa.3 | 1,047,691 | 7.8% | 1,070,737 | 7.9% | 11,377,028 | 84.3% | 13,495,456 |
| T×M.20dpa.1 | 883,080 | 7.4% | 913,776 | 7.7% | 10,057,022 | 84.8% | 11,853,878 |
| T×M.20dpa.2 | 930,008 | 7.5% | 952,974 | 7.7% | 10,490,198 | 84.8% | 12,373,180 |
| T×M.20dpa.3 | 1,015,130 | 7.8% | 1,032,388 | 7.9% | 10,992,174 | 84.3% | 13,039,692 |
| **All $F_1$s** | **11,332,177** | **7.5%** | **11,657,761** | **7.7%** | **127,649,012** | **84.7%** | **150,638,950** |

**Supplementary Table 3. Differential expression analyses of 28,716 genes containing allelic SNPs.**

| Analysis | Sample 1 | Sample 2 | 1≠2 | 1>2 | 1<2 | Chi-squared test *P*-value |
|---|---|---|---|---|---|---|
| Between parents (*A*) | Maxxa.10dpa | TX2094.10dpa | 5168 | 2715 | 2453 | 0.0003 |
| | Maxxa.20dpa | TX2094.20dpa | 3528 | 1916 | 1612 | 0.0000 |
| Between alleles in M×T (*B*) | Maxxa.M×T.10dpa | TX2094.M×T.10dpa | 1965 | 652 | 1313 | 0.0000 |
| | Maxxa.M×T.20dpa | TX2094.M×T.20dpa | 1190 | 320 | 870 | 0.0000 |
| Between alleles in T×M (*B*) | Maxxa.T×M.10dpa | TX2094.T×M.10dpa | 1827 | 563 | 1264 | 0.0000 |
| | Maxxa.T×M.20dpa | TX2094.T×M.20dpa | 1378 | 405 | 973 | 0.0000 |
| Between alleles in $F_1$ | Maxxa.F1.10dpa | TX2094.$F_1$.10dpa | 3800 | 1583 | 2217 | 0.0000 |
| | Maxxa.F1.20dpa | TX2094.$F_1$.20dpa | 2888 | 1158 | 1730 | 0.0000 |
| Between reciprocal $F_1$s | M×T.10dpa | T×M.10dpa | 824 | 239 | 585 | 0.0000 |
| | M×T.20dpa | T×M.20dpa | 1 | 0 | 1 | 0.3173 |
| Maxxa vs M×T | Maxxa.10dpa | M×T.10dpa | 3580 | 1739 | 1841 | 0.0882 |
| | Maxxa.20dpa | M×T.20dpa | 520 | 200 | 320 | 0.0000 |
| Maxxa vs T×M | Maxxa.10dpa | T×M.10dpa | 2536 | 1114 | 1422 | 0.0000 |
| | Maxxa.20dpa | T×M.20dpa | 1586 | 762 | 824 | 0.1195 |
| TX2094 vs M×T | TX2094.10dpa | M×T.10dpa | 3895 | 1697 | 2198 | 0.0000 |
| | TX2094.20dpa | M×T.20dpa | 1290 | 564 | 726 | 0.0000 |
| TX2094 vs T×M | TX2094.10dpa | T×M.10dpa | 2129 | 1018 | 1111 | 0.0438 |
| | TX2094.20dpa | T×M.20dpa | 5091 | 2323 | 2768 | 0.0000 |
| Maxxa vs $F_1$ | Maxxa.10dpa | $F_1$.10dpa | 4210 | 1995 | 2215 | 0.0007 |
| | Maxxa.20dpa | $F_1$.20dpa | 1312 | 538 | 774 | 0.0000 |
| TX2094 vs $F_1$ | TX2094.10dpa | $F_1$.10dpa | 3372 | 1354 | 2018 | 0.0000 |
| | TX2094.20dpa | $F_1$.20dpa | 4661 | 1822 | 2839 | 0.0000 |
| Maxxa allele expression between reciprocal $F_1$s | Maxxa.M×T.10dpa | Maxxa.T×M.10dpa | 85 | 15 | 70 | 0.0000 |
| | Maxxa.M×T.20dpa | Maxxa.T×M.20dpa | 2 | 2 | 0 | 0.1573 |
| TX2094 allele expression between reciprocal $F_1$s | TX2094.M×T.10dpa | TX2094.T×M.10dpa | 74 | 17 | 57 | 0.0000 |
| | TX2094.M×T.20dpa | TX2094.T×M.20dpa | 0 | 0 | 0 | n/a |

**Supplementary Table 4. Weighted co-expression gene network and subnetwork density.**

| Network | Gene number | TX2094 | Maxxa |
|---|---|---|---|
| Whole | 63665 | 0.00912485 | 0.00608408 |
| Non-RD | 26161 | 0.0087424 | 0.00595536 |
| RD | 1655 | 0.01897275 | 0.01897275 |
| *Cis*_only | 513 | 0.01898896 | 0.00820015 |
| *Cis&trans* | 841 | 0.01807372 | 0.01146843 |
| *Trans*_only | 301 | 0.03144944 | 0.01262072 |

**Supplementary Note 1. Sequence variation in association with regulatory evolution.**

To understand the genetic basis of *cis* divergence, we utilized genomic sequences for the two parental lines, Maxxa and TX2094 (SRA accession number SRR617482 and SRR3560138-3560140), to characterize genetic variants within a 2-kb promoter region upstream of the transcription start site. We asked whether *cis* regulatory divergence is mirrored in sequence variation in the gene promoter regions. A total of 340,642 SNPs (in 53,252 genes) and 88,137 indels (in 40,218 genes) were identified. As shown in Supplementary Figure 4, RD genes with *cis* divergence contain more SNPs and indels than genes without *cis* regulatory divergence (*cis*-only or *cis*+*trans* > *trans*-only; Duncan's multiple range test $P < 0.05$), and *trans*-only RD genes showed no significant difference from non-RD genes.

We next examined whether regulatory divergence was also associated with evolutionary rates (dN and dS) in coding regions. Because of the small values of dN (peak at 0.0011) and dS (peak at 0.0026), the calculation of dN/dS is subject to stochastic variance of ratios of small numbers, and was omitted in the following analysis. Both the distributions of dN and dS were significantly different between RD and non-RD genes (Kolmogorov–Smirnov test $P < 0.01$). *Cis*-only and *trans*-only genes tend to display higher substitution rate (dS = 0.0063-0.0070 and dN = 0.0028-0.0037) than those exhibiting *cis+trans* divergence and non-RD genes (dS = 0.0029-0.0035 and dN = 0.0019-0.0021; Duncan's multiple range test $P < 0.05$; Supplementary Figure 4). It was expected that *cis*+*trans* regulated genes were as conserved as non-RD genes, considering the stabilizing, antagonistic effects of co-existing *cis* and *trans* variants to preserve expression levels. However, further study is required to understand how the observed higher substitution rates of *cis*-only and *trans*-only genes relate to selection. Overall, however, the data show that RD genes with *cis*-only variants generally evolve faster with a higher substitution rate in both promoter and coding regions.

When comparing sequence divergence between corresponding At and Dt homoeologs, modest Pearson correlations of 0.15-0.38 resulted, with the highest correlation in dN and the lowest in promoter indels. Comparable amounts of promoter and coding sequence change under domestication were also observed, except that Dt promoters accumulated more SNPs than did At promoters (6.4 *vs*. 5.7 SNPs on average within 2-kb upstream of the annotated transcription start sites; Student's t-test $P < 0.05$). This slightly higher number of promoter SNPs is not associated with any particular type of *cis* and *trans* regulatory divergence.

**Supplementary Note 2. Functional implications of key TF RD genes in Maxxa versus TX2094 GRNs.**

Of the 53 RD TFs in Figure 5, 33 have more predicted TGs in Maxxa, as represented by bigger node size, than their counterparts in TX2094. For example, 2,786 and 324 TGs were predicted for node 1 (Gohir.A01G033500, response regulator RR1) in Maxxa and TX2094, respectively, which is a *trans*-only gene with increased expression following domestication. This regulator gene functions in the cytokinin signaling pathway and auxin biosynthesis, and is critical for root and shoot development in *Arabidopsis* [1,2]. In vicinity of this hub gene in Maxxa, node 6 (Gohir.D10G215300, a POWERDRESS-like MYB), 7 (Gohir.A05G132500.1, GLABRA2), 12

(Gohir.D10G033800, MIKC_MADS family protein) and 27 (Gohir.D06G209500, homeobox-1) were also predicted to have more TGs in domesticated fibers; in contrast, fewer TGs were predicted for node 45 (Gohir.D05G08900 0, TANDEM ZINC FINGER PROTEIN 9). The regulatory link in the vicinity of node 7 are of particular interest, as GLABRA2 (GL2) is a key regulator downstream of the GL1/GL3/EGL3/TTG1 core complex in the *Arabidopsis* trichome development pathway [3]. Previously, a cotton GL2-like homolog *GhHOX3* has been found to play a central role in controlling cotton fiber elongation, which represents a basal and highly conserved component of the fiber developmental program [4]; it remains an open question whether and how human-mediated selection has acted on the core regulators of trichome development such as GL2.

*Trans*-only node 41 (Gohir.D03G133500) and *cis*-only node 43 (Gohir.A03G034800) are a pair of homoeologous genes that encode ethylene-insensitive-3 family proteins, which are known to initiate downstream transcriptional cascades for ethylene response (Figure 5 and Supplementary Data 2). It has been shown that exogenously applied ethylene promotes fiber cell expansion by increasing the expression of sucrose synthase, tubulin, and expansin genes, and fiber growth is suppressed by applying the ethylene biosynthetic inhibitor L-(2-aminoethoxyvinyl)-glycine [5]. A higher expression of Dt than At was consistently observed ($\log_2$(At/Dt) = -3.3 and -3.9 in Maxxa, -1.0 and -1.3 in TX2094 at 10 and 20 dpa, respectively; all with adjusted $P < 0.05$). In the Maxxa network, a much smaller number of TGs were inferred for both nodes 41 and 43 than in TX2094 (41 – 599 Maxxa TGs *vs*. 769 TX2094 TGs, 43 – 338 *vs*. 1526; Supplementary Data 2). In the vicinity of this pair of homoeologs, nodes 6 (Gohir.D10G215300, Duplicated homeodomain-like PWR superfamily protein), 13 (Gohir.A13G064000, FAR1-related sequence 5), 27 (Gohir.D06G209500, homoeobox-1), 28 (Gohir.A13G001500, bZIP transcription factor), 45 (Gohir.D05G089000, zinc finger CCCH-type family protein), and 47 (Gohir.D07G051500, auxin response factor 2) were found in the TX2094 GRN (Figure 5b), whereas nodes 6, 27, and 45 became detached in the Maxxa GRN while still remaining interconnected among themselves (Figure 5c). Although such gain and loss of predicted links awaits experimental validation to elucidate the underlying molecular mechanisms, these results demonstrate the power of GRNs and show that the integrated analysis with evolutionary analysis of *cis* and *trans* regulatory changes can help to identify candidate genes that may have been primary targets of selection during cotton fiber domestication. Other such candidates include three genes also detected in the QTL study [6] (also see Supplementary Data 2 column of fiber QTL genes), i.e., node 3 (Gohir.A08G027700, zinc finger CCCH-type/C3HC4-type family protein), 32 (Gohir.D04G041000, homeobox protein 31), and 50 (Gohir.A08G156400, a bHLH DNA-binding family protein MYC2), and one cell-wall synthesis related gene, node 48 (Gohir.D12G242500, NAC domain transcriptional regulator superfamily protein).

## Supplementary References


1 Waldie, T. & Leyser, O. Cytokinin targets auxin transport to promote shoot branching. *Plant Physiol.* **177**, 803-818 (2018).

2 Xie, M. *et al.* A B-ARR-mediated cytokinin transcriptional network directs hormone cross-regulation and shoot development. *Nat. Commun.* **9**, 1604 (2018).

3 Hulskamp, M. Plant trichomes: A model for cell differentiation. *Nat. Rev. Mol. Cell Biol* **5**, 471-480 (2004).

4 Shan, C. M. *et al.* Control of cotton fibre elongation by a homeodomain transcription factor *GhHOX3*. *Nat. Commun.* **5**, 5519 (2014).

5 Shi, Y. H. *et al.* Transcriptome profiling, molecular biological, and physiological studies reveal a major role for ethylene in cotton fiber cell elongation. *Plant Cell* **18**, 651-664 (2006).

6 Grover, C. E. *et al.* Genetic analysis of the transition from wild to domesticated cotton (*G. hirsutum*). Preprint at https://www.biorxiv.org/content/10.1101/616763v1 (2019).
