# Supplementary material for: Unraveling cis and trans regulatory evolution during cotton domestication
Source: Nat Commun. 2019 Nov 27;10:5399. doi: 10.1038/s41467-019-13386-w (PMC6881400; doi:10.1038/s41467-019-13386-w)
Supplement: Supplementary file 3 — Reporting Summary [file 41467_2019_13386_MOESM3_ESM.pdf]

nature research

Corresponding author(s): NCOMMS-19-24197B

Last updated by author(s): Nov 3, 2019

Reporting Summary

Nature Research wishes to improve the reproducibility of the work that we publish. This form provides structure for consistency and transparency in reporting. For further information on Nature Research policies, see Authors & Referees and the Editorial Policy Checklist.

Statistics

For all statistical analyses, confirm that the following items are present in the figure legend, table legend, main text, or Methods section.

n/a	Confirmed	
☐	☒	The exact sample size (*n*) for each experimental group/condition, given as a discrete number and unit of measurement
☐	☒	A statement on whether measurements were taken from distinct samples or whether the same sample was measured repeatedly
☐	☒	The statistical test(s) used AND whether they are one- or two-sided<br>*Only common tests should be described solely by name; describe more complex techniques in the Methods section.*
☒	☐	A description of all covariates tested
☐	☒	A description of any assumptions or corrections, such as tests of normality and adjustment for multiple comparisons
☐	☒	A full description of the statistical parameters including central tendency (e.g. means) or other basic estimates (e.g. regression coefficient) AND variation (e.g. standard deviation) or associated estimates of uncertainty (e.g. confidence intervals)
☐	☒	For null hypothesis testing, the test statistic (e.g. *F*, *t*, *r*) with confidence intervals, effect sizes, degrees of freedom and *P* value noted<br>*Give P values as exact values whenever suitable.*
☒	☐	For Bayesian analysis, information on the choice of priors and Markov chain Monte Carlo settings
☒	☐	For hierarchical and complex designs, identification of the appropriate level for tests and full reporting of outcomes
☒	☐	Estimates of effect sizes (e.g. Cohen's *d*, Pearson's *r*), indicating how they were calculated

Our web collection on statistics for biologists contains articles on many of the points above.

Software and code

Policy information about availability of computer code

Data collection	RNA-seq data was generated using Illumina - HiSeq 2500 and its software.
Data analysis	We used TrimeGlore (v0.4.3) and Cutadapt (v1.11) to filter and trim raw RNA-seq reads, Bowtie2(v2.3.4) and samtools (1.9) for read mapping and processing, and HyLiTE(v1.0) for SNP calling and read classification in F1 hybrids. For analyzing DNA data, we used SOAPnuke (v1.5.2) for quality filtering and trimming, bwa (v0.7.10) for mapping, GATK (v3.5) and FreeBayes (v1.0.2) for variant calling, snpEff (v4.2) for SNP annotation, and PAML (v4.9) for estimating evolution rates. Identification of homologous gene pairs was done using OrthoFinder (v2.1.2) and MSCanX (2017-4–3). Following analyses were performed in R environment (3.5.0): DESeq2 (1.14.1) used to perform normalization and differential gene expression analyses; WGCNA (1.64) for co-expression network analysis; GENIE3 for constructing regulatory gene network (1.6.0); clusterProfiler (3.12.0) for GO enrichment and GSEA analysis; ggplot2 (3.1.1) for making plots. All custom scripts are deposited in GitHub (https://github.com/Wendellab/CisTransRegulation).

For manuscripts utilizing custom algorithms or software that are central to the research but not yet described in published literature, software must be made available to editors/reviewers. We strongly encourage code deposition in a community repository (e.g. GitHub). See the Nature Research guidelines for submitting code & software for further information.

Data

Policy information about availability of data

All manuscripts must include a data availability statement. This statement should provide the following information, where applicable:

- Accession codes, unique identifiers, or web links for publicly available datasets
- A list of figures that have associated raw data
- A description of any restrictions on data availability

The RNA-seq data sets generated in the current study are available in the NCBI Short Read Archive (PRJNA529497), with accession numbers summarized in Supplementary Table 1. The genomic DNA data that support the findings of this study are available in NCBI Sequence Read Archive (SRR617482 and

SRR3560138-3560140).

Field-specific reporting

Please select the one below that is the best fit for your research. If you are not sure, read the appropriate sections before making your selection.

☒ Life sciences ☐ Behavioural & social sciences ☐ Ecological, evolutionary & environmental sciences

For a reference copy of the document with all sections, see nature.com/documents/nr-reporting-summary-flat.pdf

Life sciences study design

All studies must disclose on these points even when the disclosure is negative.

Sample size	Sample size per group or condition was determined based on the minimum number of biological replicates required to perform differential expression analysis as per software tools used and previously published literature.
Data exclusions	Samples were excluded when they displayed poor correlation between biological replicates; clustering with samples from other conditions suggests mislabeling during sample collection or the RNA-seq library preparation stages.
Replication	Findings were consistent between biological replicates and different sequencing plates/batches.
Randomization	Order of sample collection, RNA extraction, processing for library preparation and sequencing were processed in multiple batches, kind of randomization in itself, but following stringent standardized protocols.
Blinding	No blinding took place. To alleviate any complications from non-blinded analyses all samples were analyzed simultaneously in the same manner regardless of their condition/origin.

Reporting for specific materials, systems and methods

We require information from authors about some types of materials, experimental systems and methods used in many studies. Here, indicate whether each material, system or method listed is relevant to your study. If you are not sure if a list item applies to your research, read the appropriate section before selecting a response.

Materials & experimental systems

n/a	Involved in the study	
☒	☐	Antibodies
☒	☐	Eukaryotic cell lines
☒	☐	Palaeontology
☒	☐	Animals and other organisms
☒	☐	Human research participants
☒	☐	Clinical data

Methods

n/a	Involved in the study	
☒	☐	ChIP-seq
☒	☐	Flow cytometry
☒	☐	MRI-based neuroimaging
