# Supplementary material for: Unraveling cis and trans regulatory evolution during cotton domestication
Source: Nat Commun. 2019 Nov 27;10:5399. doi: 10.1038/s41467-019-13386-w (PMC6881400; doi:10.1038/s41467-019-13386-w)
Supplement: Supplementary file 4 — Description of Additional Supplementary Files [file 41467_2019_13386_MOESM4_ESM.docx]

**Additional Description of Additional Supplementary Files**

File name: Supplementary Data 1.

Description: Comparison of *cis* and *trans* effects using both the standard and cross-replicate approaches.

File name: Supplementary Data 2.

Description: Table of 1655 regulatory divergent (RD) genes.

File name: Supplementary Data 3.

Description: Cross-tabulation of regulatory and inheritance categories.

File name: Supplementary Data 4.

Description: GO enrichment analysis of 55,551 fiber expressed genes against of the universe of 66,610 cotton genes.

File name: Supplementary Data 5.

Description: GO enrichment analysis of Maxxa vs TX2094 DE genes each at 10 dpa and 20 dpa against of the universe of 55,551 fiber expressed genes.

File name: Supplementary Data 6.

Description: GO enrichment analysis of regulatory divergent (RD) genes against of the universe of 27815 genes surveyed.
