# Supplementary material for: Unraveling cis and trans regulatory evolution during cotton domestication
Source: Nat Commun. 2019 Nov 27;10:5399. doi: 10.1038/s41467-019-13386-w (PMC6881400; doi:10.1038/s41467-019-13386-w)
Supplement: Supplementary file 2 — Peer Review File [file 41467_2019_13386_MOESM2_ESM.pdf]

Reviewers' comments:

Reviewer #1 (Remarks to the Author):

This is a very nice paper, well written and readable despite great (and necessary) detail on a topic that is by no means always easy to grasp. It adds significant new elements to the increasingly rich interdigitated dual stories of polyploidy and domestication in cotton that the Wendel lab has spearheaded for decades. Cotton is a model for studying both phenomena, given the availability of known, extant progenitors—both of the two homoeologous genomes that comprise G. hirsutum and of wild tetraploids. Both resources are used to great advantage here, as the authors tease apart the interlocking elements of cotton's evolution and domestication history, focusing on the biology of the fibers that make it such an important crop species. The work is original, well-conceived and executed, reports novel results that are of significance for several audiences, and, as already noted, is well-written.

I really don't have significant general comments about it. I do have numerous mostly small comments that I hope will improve the clarity of the paper if they are addressed.

Comments by line number:

153-154. How Fig. 1A differs from Fig. 2A needs to be clarified in the legends.

The 1A legend says: "The percentage of genes assigned to each of the seven possible categories of regulatory evolution is shown next to each category." All 7 categories are shown.

The 2A legend says: "Numbers and percentages of genes for the regulatory categories I-IV ... ".

According to this the 2A numbers should be a subset of 1A values. The percentages in 2A should refer to the proportion of categories I-IV so will be higher than I-IV percentages in 1A, but should be proportional. But they aren't ... or are they?

Actually, the problem seems to be that this is overstated--it is true that a higher proportion is consistently observed, as it says, but the difference is not high; it is just more pronounced when the unaffected or ambiguous categories are removed.

156. In Fig. 1, cis-only (I) is 1.1-2.2%; trans-only (II) is 0.9-1.1%. I'm not exactly sure what the ranges mean, but there is at most a 2x difference.

However, this refers to Fig. 2, not Fig. 1.

160-161. In Fig. 1, category III (enhancing) is 0.1%; category IV (compensating) is 0.4-0.7%. This is a 4-7x difference, not an 8-20x difference as stated here.

173-176. I don't understand this. Why? Because cis is the only effect that can be directly inferred? Regardless whether I'm correct, there should be a "because" statement added here.

212. In the expression "IV/V", does "/" really mean "divided by", which is how I'd usually interpret this? Shouldn't it be (IV+V)?

214-215. This sort of statement more properly belongs in Discussion than in Results. It is not a "result" but rather an inference drawn from the result.

226-228. I find this to be either self-evident or cryptic--I'm not sure which. In either case, it may be better in Discussion.

278. In Fig. 4B legend, why is a percentage only given for TX RD/Bias (65.3%), but not for Maxxa (or for any other cell)?

293. In Fig. S3, the label should be "Homoeolog ratio" (not "ration").

314. Concerning Fig. S4, I think it would be visually superior to have the same order of categories in all subfigures so that the reader can see at a glance which categories are statistically different. Grouping by statistical significance requires the reader to do more processing, and is actually misleading at first glance, because the expectation (from almost all literature) is that the order will be the same, not changed to reflect statistical groupings.

322. Presumably this should be "substitution rate" and not mutation rate, since this is not a mutation accumulation experiment.

325-328. There is something wrong with this sentence. There needs to be some conclusion to the "While we expected that” clause. What it was that was expected is never stated!

329. Again, there is nothing known about the underlying mutation rate. What is observed are substitutions accepted though selection.

352. This implies a time component (to the analysis?). Maxxa is not younger than TX2094--both are modern plants.

Perhaps my problem is that I'm not visualizing the comparison properly? Are the networks different between MAXXA and TX? Where does the reader get this information? I was initially envisioning the same networks being compared in the two accessions (since they are conspecific). There is no indication here that domestication has altered the entire regulatory wiring of fiber production--perhaps that needs to be stated. Fig. 5 presumably gives at least part of the answer, but is not referred to until later.

353. I'm not sure how to interpret this statement. Does it mean that completely different networks were involved in domestication? But these are also "fiber" networks. Is the distinction meant to be between "wild fiber" networks vs. "domesticated fiber" networks? If so, what is the difference?

395. 5B/C legend mentions WGCNA modules. Where are these listed? There seems to be no information about WGNA analyses, other than summaries. There is a list of supplementary tables in the ms, but the only supplementary table I can find in the supplementary files I downloaded is Table S7.

396-397. Ref. 79 is listed as "in preparation", which hardly qualifies as a "previous report"!

463-464. This is very awkward. The first clause is a stand-alone declarative sentence; the second clause is not. It should either be (something like):

Important roles of cis- and trans- regulatory changes ... and the likely importance of polyploidy

OR

Both cis and trans regulatory changes, as well as polyploidy, have played important roles during cotton domestication

488-492. This seems simplistic and overgeneralized to me, and also may be more applicable to animals than to plants (given the more pronounced canonical enhancer structure in animals). I would

be more comfortable if this set of statements had references of its own, so that it would be clear that these are conclusions from the literature. But even then, I wonder at the math involved. What constitutes a trans factor, for example for the purposes of this statement? Are only TFs being considered here, as seems to be the case throughout the empirical parts of the paper? Is the math underlying the statements refer to each individual gene, saying that cis factors are confined to the proximal noncoding region of that gene, whereas the gene's expression could be affected by several trans factors (even if only confined to TFs) scattered throughout the genome--this would indeed increase the mutational target size in favor of trans factors over cis. Alternatively, if ~50K structural genes are being considered as possible candidates for cis mutations, each with 1-2 kb of potential cis regulatory target sequence, vs. a few thousand TFs, then perhaps the math is reversed. Some additional explanation of what is meant here would help.

520. It might be helpful to remind readers about genome bias in expression of homoeologues, relative to genome size in G. hirsutum. The prediction would be that the genome with a higher density of TEs would be less expressed, in general. Whether that is the larger genome (size difference due to more TEs, which I'd expect) or the smaller (same number of TEs in a smaller genomic space, which seems unlikely) in this case might be mentioned.

525-527. This would seem like a good place to reference Hu et al. (2019; ref. 57).

557-559. This begs for some additional comment! Is there any evidence in cotton? How would you tell? Weaker omega values? What's the definition of "essential genes", when pretty much all genes are under purifying selection? Could this refer to the pan-genome? To homoeologous genes with strongly biased expression? The latter would be a nice transition to the next subheading topic!

571-574. This should be spelled out more clearly: "... creates the opportunity for novel trans interactions that affect both homoeologs equally, resulting in changes to overall transcription but not homoeolog ratio." The reasoning wasn't immediately apparent, to me, at least. And maybe I still don't have it right!

583-584. This seems out of date to me, harkening back to a few years ago (e.g., Garsmeur et al. 2014) when it was accepted (by many) that all allopolyploids showed subgenome bias. Cotton is an example, certainly, and one of the two references pertains to cotton. But a more nuanced view it is now widely accepted--that the issue is progenitor genome divergence, not mode of polyploid origin (see Capsella). I think "likely most" here is an overstatement--the sample size is still too small. Why not something like "and allopolyploids whose genomes were formed by diploids with different regulatory regimes"? Leaving out "allo" might even be considered, in consideration of ASE bias in heterozygous autopolyploids such as Medicago sativa.

590. It would be good to refer to Fig. 4C.

615-616. What about the following papers in cotton? Is there nothing to discuss from those relevant to this work?

Wang M, Wang P, Lin M, Ye Z, Li G, Tu L, Shen C, Li J, Yang Q, Zhang X. 2018. Evolutionary dynamics of 3D genome architecture following polyploidization in cotton. Nat Plants 4(2): 90-97.

Wang M, Tu L, Lin M, Lin Z, Wang P, Yang Q, Ye Z, Shen C, Li J, Zhang L, et al. 2017. Asymmetric subgenome selection and cis-regulatory divergence during cotton domestication. Nat Genet 49(4): 579-587.

The 2017 paper, which is cited early in the Results here, has this quote: “By comparing overlaps with domestication signals, we identified 620 homoeologous pairs that have been subjected to domestication selection in the At or Dt (192 in the At and 428 in the Dt) and only 34 homoeologous

pairs with selection signals in both subgenomes". This seems relevant here, as does other material on identification of cis-regulatory elements. I'm surprised there is no discussion of it.

636. Insights from new technologies are mentioned, so I would think this could be a good place to cite the Wang et al. papers (again).

Reviewer #2 (Remarks to the Author):

The authors estimate the contribution of cis and trans effects to regulatory divergence between wild and domesticated cotton. They summarize the prevalence and direction of cis and trans effects to understand their contribution to the domestication of cotton. In general I think the work is interesting, but is probably better suited to a journal with a more specialized readership.

The number one thing I think needs to be addressed here are the issues brought up by Fraser (2019) considering the appearance of compensatory cis trans differences and stabilizing selection. Unless the authors estimate cis and trans from separate replicates, this shouldn't be part of their conclusions. To be clear, I don't require that they do this, only that compensatory changes/stabilizing selection are not considered a conclusion from this work.

The abstract and laundry-listy and hard to follow. Line 20, QTLs are mentioned out of nowhere. Line 23, why do you propose that? Do non-polyploids have less trans regulatory contribution? What are you comparing to?

56 I don't know that that logic follows

70-80 The logic here doesn't really flow. 1. How to estimate cis/trans 2. In other species cis becomes more dominant with time 3. In plants both seem to be important, also in response to environment? 4. How humans effect cis and trans is not explored

105 discern and parse?

118 what does dpa stand for? Ah, I see it is brought up on line 141, maybe correct that

169 As aforementioned

The use of categories is not very helpful, rather than expecting the reader to remember what category II is, or saying category II (trans only) why not just say what they are – 'trans only' 'cis only' etc. Again the point here around line 209 and the appearance of compensatory evolution. Also the use of A and B is confusing, just say what they stand for rather than creating a variable, its unnecessary.

262 Yoo and Wendel? Maybe just previous work. Also, what is the significance of this?

276 What is an RD gene

271-282 Not totally sure what you trying to say here – that homeologs with expression differences in wild cotton were more likely to show additional divergence during domestication? Maybe say that more explicitly, or if I'm wrong, state what you are trying to say more clearly.

The whole section on enrichment is speculative. Line 358 – Why? Wouldn't cis genes be connected in trans to other genes, or are you presuming that cis genes are at the edge of networks? Are these subnetwork density differences significant? What is the maximum density, .012 versus .008 seems like a small difference. Line 362 I don't understand this sentence.

Functional enrichment analysis – can we get some p values?

Why was an acronym introduced in that section (GSEA) Will it ever be used again

385 Is it really necessary to introduce three new acronyms

394 How is this measured? Is it just eye balling? Why do you say smaller gene islands i.e. components? What does that communicate?

~425 The discussion of network changes is hard to follow. A summary of conclusions and a reference to the figure may be more welcome.

432 What three fiber QTL genes? Is this from a different paper?

Why are QTLs mentioned in the abstract, and then randomly in the results, when you are comparing to a different study? This needs to be clarified. In general the paper is really long and detailed, while also not being clearly written. If you want to compare to QTLs from previous papers, have a section that does this and clearly summarize the results of overlap between previous QTLs and this study. Were 45 QTLs identified, and 7 overlap with this study?

In the discssuion, again the point about stabilizing selection. In the discussion I think there should be a more succinct summary of the importance of the results, rather than a laundry list of the results again.
Again about the categories, why?
529. 534 the significant correspondence between cis only genes (category I) and additivity by inheritance
530. 535 was expected, given that in this regulatory category, parental expression ratios were maintained
531. 536 between alleles in the F1 hybrids; hence, the aggregated expression of alleles should be
532. 537 intermediate to the parental values unless there exists some form of additional regulatory
533. 538 perturbation. In the case of expression-level dominance whereby the hybrid resembles one of two
534. 539 parental states, significant correspondence was found for trans only genes (category II), as well
535. 540 as those exhibiting compensating effects of coexisting cis and trans variants (IV).
The manuscript is really long and difficult to read.
618 – how is this relevant to the current study? This whole paragraph reads like a book report rather than a summary of previous literature, and how the current study adds to that understanding.
619- Was it enriched for QTL regions? Where was this analysis? I saw one mention of QTLs overlapping at the end of a section, and there was no measure of significant enrichment??

681 Its not the BH method unless you define that acronym, and again why would you, it's the Benjamini-Hochberg method.

You define CRE in the manuscript as an acronym but never use it, ASE but you only use it one more time after defining it. GRN is redefined twice and not used that many times, GSEA is used twice and defined twice (at the same time). If you want your reader to take the effort of remembering an acronym then use them wisely.
It seems like the difference between the two genomes in size should be really interested, and more

fully explored. The paper is really long and detailed, and I'm not sure how interesting or useful a lot of the information provided is. Just because an analysis was done, does not mean it needs to be included in the paper.

Figure 2A What is the difference between compensating and compensatory? Why can you only see red, blue, purple, and maroon in these figures? If the colors apply to a, not b, and then c-f you might consider reordering the figures

Reviewer #3 (Remarks to the Author):

Review of Bao et al., Unraveling cis and trans regulatory evolution during cotton domestication.

Overview:

In this manuscript, the authors undertake a large-scale genetic cross/RNA-seq analysis to understand the nature of the regulatory divergence between wild and domesticated cotton. They find large contributions to differential expression from both cis and trans changes, often finding that the changes are in compensating directions, such that overall expression level is conserved across the strains.

Major Comments:
This is a well-executed and interesting experiment that adds to our understanding of regulatory evolution. However, I have some reservations about the statistical analyses and presentation that I think lead the manuscript to be overly definitive in its interpretation of the data collected.

My first concern is best understood when comparing class IV and V in figure 2: The "compensatory" class represents parents with no observed differential expression yet showing allele-specific differences in the hybrids. The issue this raises is that the statistical models the authors use to identify differentially-expressed genes are perfectly valid, but are intrinsically pair-wise. Hence, effects such as differences in statistical power can give rise to cases such #V, which arguably could be an instance where the difference between the parents was too slight to reject the null hypothesis but for some reason exceeded that threshold in the F1. Although I have given this as a particular example, I think the problem is general: strictly assigning genes or alleles as either differentially-expressed or not in pairwise comparisons and then using those test results to build the sort of cis-trans model used here is problematic because the inherent variation in the experiment is only considered in the pairwise comparisons, but there is also a component of variation between the parents and the F1s, such that even in a "perfect" cis case we do not expect the parental expression levels to precisely predict the F1 levels. Perhaps the authors' approach captures this problem, but it does not appear so from the methods: instead, the authors assign the part of the variance not explained by cis effects in the F1 as by definition trans effects. But the t-test used to do this, would not, to my mind, capture the fact that there are unmeasured errors in the estimates of the size of the cis effects in both parents and in the F1s. Hence this "trans" category represents true trans effects as well as simple experimental noise not captured by the authors' statistical approaches. I would strongly urge the authors to consult a statistician, if for no other reason than to refute the argument above.

A related concern that I don't think requires more analysis but needs to be at least acknowledged by the authors is that the model used assumes no interactions between cis and trans effects, allowing the separation described above. Now we know that there *are* interactions: from a simple biochemical perspective, if one cis binding site increases its affinity for a trans-factor, the excess binding of that factor to that site decreases the apparent concentration of the factor at the other allele, even if the overall concentration of the factor is unaltered. This is only an example: there are multiple ways in which biochemical systems feedback and adapt. Hence, while I think the authors' approach is acceptable for a first-pass analysis, I think the assumption on which the cis/trans differentiation is

being made should be highlighted more.

These interaction effects lead me to my final major concern: the authors' interpretation of the excess of compensating cis/trans interactions. The authors attribute the excess of compensating interactions over enhancing ones to the actions of purifying selection on gene expression levels: presumably through the fixation of compensating cis changes after a trans change or vice versa. This is a possible interpretation that is likely correct in some cases. But it ignores a very plausible null hypothesis, which is that these regulatory systems are subject to various forms of feedback (1). Feedback regulation at the mRNA or protein level will produce this same excess of compensatory changes in expression levels without a need to directly invoke selection on expression levels (2). Moreover, the feedback regulation need not be due to direct selection on expression level: it can also result from selection against noise in gene expression (3).

There are a few more minor issues with the manuscript, but I think they can more appropriately be addressed once the overall framework of the manuscript is improved with better statistical models.

References:
1. Becskei A & Serrano L (2000) Engineering stability in gene networks by autoregulation. Nature 405(6786):590-593.
2. Wagner A (2005) Robustness and Evolvability in Living Systems (Princeton University Press, Princeton, NJ).
3. Rao CV, Wolf DM, & Arkin AP (2002) Control, exploitation and tolerance of intracellular noise. Nature 420(6912):231-237.

Reviewers' comments:

Reviewer #1 (Remarks to the Author):

This is a very nice paper, well written and readable despite great (and necessary) detail on a topic that is by no means always easy to grasp. It adds significant new elements to the increasingly rich interdigitated dual stories of polyploidy and domestication in cotton that the Wendel lab has spearheaded for decades. Cotton is a model for studying both phenomena, given the availability of known, extant progenitors—both of the two homoeologous genomes that comprise G. hirsutum and of wild tetraploids. Both resources are used to great advantage here, as the authors tease apart the interlocking elements of cotton's evolution and domestication history, focusing on the biology of the fibers that make it such an important crop species. The work is original, well-conceived and executed, reports novel results that are of significance for several audiences, and, as already noted, is well-written.

I really don't have significant general comments about it. I do have numerous mostly small comments that I hope will improve the clarity of the paper if they are addressed.

> We appreciate this positive and encouraging comment, and have made all of the suggested changes listed below.

Comments by line number:

153-154. How Fig. 1A differs from Fig. 2A needs to be clarified in the legends.

The 1A legend says: "The percentage of genes assigned to each of the seven possible categories of regulatory evolution is shown next to each category." All 7 categories are shown.

The 2A legend says: "Numbers and percentages of genes for the regulatory categories I-IV ... ".

According to this the 2A numbers should be a subset of 1A values. The percentages in 2A should refer to the proportion of categories I-IV so will be higher than I-IV percentages in 1A, but should be proportional. But they aren't ... or are they?

Actually, the problem seems to be that this is overstated--it is true that a higher proportion is consistently observed, as it says, but the difference is not high; it is just more pronounced when the unaffected or ambiguous categories are removed.

> Thank you for pointing out this clarity issue. In Figure 1b, the gene percentage refers to the proportion of genes assigned to each of the 7 categories; category I-IV accounts for 4.1% of 27,816 genes (=1,152 genes) for 10 dpa MxT. In Figure 2a, the first column from left corresponds to the category I-IV genes shown in Figure 1b, and percentages here refer to the proportion of genes out of the total number of 1,152.
>
> The legend of Figure 2a, accordingly, was edited to be more clear: "**a**. Regulatory categories I-IV that exhibited parental divergence. In 10 and 20 dpa fibers from the

reciprocal F1 hybrids M×T and T×M, gene numbers and relative percentages of these four categories were shown." We also modified the color legend in Figure 2a for clarity.

Regarding the text in lines 153-154, the reviewer is correct that the actual gene numbers of category I and II are not high when considering their proportions out of 27,816 genes, but their relative importance (80%) out of the genes exhibiting parental divergence (category I-IV) is also true as stated here.

156. In Fig. 1, cis-only (I) is 1.1-2.2%; trans-only (II) is 0.9-1.1%. I'm not exactly sure what the ranges mean, but there is at most a 2x difference.

However, this refers to Fig. 2, not Fig. 1.

The ranges in Figure 1A were used to summarize the percentages obtained from four sample conditions – MxT and TxM hybrids each at 10 and 20 dpa fiber stage. The corresponding figure legend was modified for clarity. Both Figure 1a and Figure 2a support the claim of more I than II genes, so we included both in relevant text.

160-161. In Fig. 1, category III (enhancing) is 0.1%; category IV (compensating) is 0.4-0.7%. This is a 4-7x difference, not an 8-20x difference as stated here.

This is also a confusion caused by the above figure legend issues. The percentage ranges of Figure 1a should not be directly used to derive the fold-change differences, due to their meanings, as explained above. Instead, the fold-change differences were calculated for each sample condition using the gene numbers of Figure 2A (MxT 10dpa 7x, TxM 10 dpa 9.8x, MxT 20 dpa 20.2x, TxM 20 dpa19.7x), which are summarized as 7-20x.

173-176. I don't understand this. Why? Because cis is the only effect that can be directly inferred? Regardless whether I'm correct, there should be a "because" statement added here.

We rewrote this sentence as suggested: "Because the measure of ***A*** was common for MxT and TxM hybrids while each of their ***B*** values was separately inferred, we would like to point out the inference of regulatory divergence was not completely independent between the reciprocal hybrids."

212. In the expression "IV/V", does "/" really mean "divided by", which is how I'd usually interpret this? Shouldn't it be (IV+V)?

We changed "IV/V" to "IV or V", which is meant to represent "either compensating or compensatory regulation".

214-215. This sort of statement more properly belongs in Discussion than in Results. It is not a "result" but rather an inference drawn from the result.

Agreed; we moved it to Discussion.

226-228. I find this to be either self-evident or cryptic--I'm not sure which. In either case, it may be better in Discussion.

> We deleted this statement.

278. In Fig. 4B legend, why is a percentage only given for TX RD/Bias (65.3%), but not for Maxxa (or for any other cell)?

> Thank you for pointing out this mistake. The single percentage was removed in this revision to keep the contingency table clear, using only numbers.

293. In Fig. S3, the label should be "Homoeolog ratio" (not "ration").

> Corrected.

314. Concerning Fig. S4, I think it would be visually superior to have the same order of categories in all subfigures so that the reader can see at a glance which categories are statistically different. Grouping by statistical significance requires the reader to do more processing, and is actually misleading at first glance, because the expectation (from almost all literature) is that the order will be the same, not changed to reflect statistical groupings.

> We fixed this figure to ensure the same order of categories on the X-axis.

332. Presumably this should be "substitution rate" and not mutation rate, since this is not a mutation accumulation experiment.

> Agreed; we have replaced all the uses of "mutation rate" with "substitution rate".

325-328. There is something wrong with this sentence. There needs to be some conclusion to the "While we expected that" clause. What it was that was expected is never stated!

> We rewrote this sentence as "It was expected that *cis*+*trans* regulated genes were as conserved as non-RD genes, considering the stabilizing, antagonistic effects of co-existing *cis* and *trans* variants to preserve expression levels. However, further study is required to understand whether and how the observed higher substitution rates of *cis*-only and *trans*-only genes relate to selection."

329. Again, there is nothing known about the underlying mutation rate. What is observed are substitutions accepted though selection.

> Corrected as "substitution rate".

352. This implies a time component (to the analysis?). Maxxa is not younger than TX2094--both are modern plants.

Perhaps my problem is that I'm not visualizing the comparison properly? Are the networks different between MAXXA and TX? Where does the reader get this information? I was initially envisioning the same networks being compared in the two accessions (since they are conspecific). There is no indication here that domestication has altered the entire regulatory wiring of fiber production--perhaps that needs to be stated. Fig. 5 presumably gives at least part of the answer, but is not referred to until later.

We edited this paragraph to clarify that a WGCNA network was generated independently each for Maxxa and TX2094, which enabled the direct comparison of network hub genes between representatives of domesticated and wild cotton, respectively. The reviewer is correct that TX2094 and Maxxa are both modern plants, so we changed the misleading phrase "no longer" to be "not".

353. I'm not sure how to interpret this statement. Does it mean that completely different networks were involved in domestication? But these are also "fiber" networks. Is the distinction meant to be between "wild fiber" networks vs. "domesticated fiber" networks? If so, what is the difference?

As mentioned above, two separate wild and domesticated fiber networks were generated, which exhibited different network properties for RD genes: highly connected as hubs in TX2094, but not in Maxxa. We hope the revised text addresses this concern.

395. 5B/C legend mentions WGCNA modules. Where are these listed? There seems to be no information about WGNA analyses, other than summaries. There is a list of supplementary tables in the ms, but the only supplementary table I can find in the supplementary files I downloaded is Table S7.

The generation of WGCNA networks was now added in the results section "Co-expression network analysis implicates functional association of regulatory genes", and the assignment of consensus WGCNA modules is now provided in Table S4 and is described in the Methods.

In our original submission, the supplementary tables were provided as multiple sheets within a single excel file; we have submitted them as individual files in this revision.

396-397. Ref. 79 is listed as "in preparation", which hardly qualifies as a "previous report"!

We edited this sentence to be "This increased level of interconnectivity in domesticated versus wild cotton is consistent with the previous report in seed development [68], as well as a separate study of fiber co-expression networks [80]."

463-464. This is very awkward. The first clause is a stand-alone declarative sentence; the second clause is not. It should either be (something like):

Important roles of cis- and trans- regulatory changes ... and the likely importance of polyploidy

OR

Both cis and trans regulatory changes, as well as polyploidy, have played important roles during cotton domestication

> This subtitle now reads as "Both *cis* and *trans* regulatory changes have played important roles during cotton domestication, likely related to polyploidy".

488-492. This seems simplistic and overgeneralized to me, and also may be more applicable to animals than to plants (given the more pronounced canonical enhancer structure in animals). I would be more comfortable if this set of statements had references of its own, so that it would be clear that these are conclusions from the literature. But even then, I wonder at the math involved. What constitutes a trans factor, for example for the purposes of this statement? Are only TFs being considered here, as seems to be the case throughout the empirical parts of the paper? Is the math underlying the statements refer to each individual gene, saying that cis factors are confined to the proximal noncoding region of that gene, whereas the gene's expression could be affected by several trans factors (even if only confined to TFs) scattered throughout the genome--this would indeed increase the mutational target size in favor of trans factors over cis. Alternatively, if ~50K structural genes are being considered as possible candidates for cis mutations, each with 1-2 kb of potential cis regulatory target sequence, vs. a few thousand TFs, then perhaps the math is reversed. Some additional explanation of what is meant here would help.

> Agreed. The underlying mechanism is much more complicated and a widely accepted synthesis is still lacking, so we deleted these speculative statements.

520. It might be helpful to remind readers about genome bias in expression of homoeologues, relative to genome size in G. hirsutum. The prediction would be that the genome with a higher density of TEs would be less expressed, in general. Whether that is the larger genome (size difference due to more TEs, which I'd expect) or the smaller (same number of TEs in a smaller genomic space, which seems unlikely) in this case might be mentioned.

> Agreed. We inserted the following sentence: "It is worth noting that different parental TE loads and their relative distribution between subgenomes have been the most popular explanation for biased homoeolog expression and biased genome fractionation [104, 105, 106, 107], which likely further complicates regulatory circuits in allopolyploids."

525-527. This would seem like a good place to reference Hu et al. (2019; ref. 57).

> Done.

557-559. This begs for some additional comment! Is there any evidence in cotton? How would you tell? Weaker omega values? What's the definition of "essential genes", when pretty much all genes are under purifying selection? Could this refer to the pan-genome? To homoeologous genes with strongly biased expression? The latter would be a nice transition to the next subheading topic!

We did consistently observe more transgressive up-regulated than down-regulated genes (Figure 3c and Table S6). In Schaefe et al, gene essentiality was defined by low fitness in knock-out experiments of the given gene. We edited these sentences to improve clarity.

571-574. This should be spelled out more clearly: "... creates the opportunity for novel trans interactions that affect both homoeologs equally, resulting in changes to overall transcription but not homoeolog ratio." The reasoning wasn't immediately apparent, to me, at least. And maybe I still don't have it right!

Edited as suggested.

583-584. This seems out of date to me, harkening back to a few years ago (e.g., Garsmeur et al. 2014) when it was accepted (by many) that all allopolyploids showed subgenome bias. Cotton is an example, certainly, and one of the two references pertains to cotton. But a more nuanced view it is now widely accepted--that the issue is progenitor genome divergence, not mode of polyploid origin (see Capsella). I think "likely most" here is an overstatement--the sample size is still too small. Why not something like "and allopolyploids whose genomes were formed by diploids with different regulatory regimes"? Leaving out "allo" might even be considered, in consideration of ASE bias in heterozygous autopolyploids such as Medicago sativa.

We wonder if the reviewer is confusing “homoeolog expression bias”, i.e., unequal expression of two homoeologs, with “subgenome bias” or “genome dominance”. The latter terms, as used by Garmeur et al (2014), refer to nonequivalence of two (or more) subgenomes with respect to the overall level of gene loss following polyploid formation. In our case, homoeolog expression bias refers to the common phenomenon whereby there is unequal expression of the two (or more) duplicated copies (= homoeologs) of any given gene in a polyploid, in one or more tissues.

590. It would be good to refer to Fig. 4C.

Done.

615-616. What about the following papers in cotton? Is there nothing to discuss from those relevant to this work?

Wang M, Wang P, Lin M, Ye Z, Li G, Tu L, Shen C, Li J, Yang Q, Zhang X. 2018. Evolutionary dynamics of 3D genome architecture following polyploidization in cotton. Nat Plants 4(2): 90-97.

Wang M, Tu L, Lin M, Lin Z, Wang P, Yang Q, Ye Z, Shen C, Li J, Zhang L, et al. 2017. Asymmetric subgenome selection and cis-regulatory divergence during cotton domestication. Nat Genet 49(4): 579-587.

The 2017 paper, which is cited early in the Results here, has this quote: “By comparing overlaps with domestication signals, we identified 620 homoeologous pairs that have been subjected to domestication selection in the At or Dt (192 in the At and 428 in the Dt) and only 34 homoeologous pairs with selection signals in both subgenomes”. This seems relevant here, as

does other material on identification of cis-regulatory elements. I'm surprised there is no discussion of it.

> We added the following discussion: "Recently, global analyses of DNase I–hypersensitive sites and 3D genome architecture in *G. hirsutum* were used to profile promoter and distant *cis* regulatory elements, respectively [61, 88]; these elements were intersected with selection sweeps identified from a variation map of 352 wild and domestication cottons to demonstrate the domestication effect on *cis* divergence and transcription regulation [60]. These examples shed light on how integrated analysis from various data sources might lead to validation of candidate RD genes."

636. Insights from new technologies are mentioned, so I would think this could be a good place to cite the Wang et al. papers (again).

> Done.

Reviewer #2:

The authors estimate the contribution of cis and trans effects to regulatory divergence between wild and domesticated cotton. They summarize the prevalence and direction of cis and trans effects to understand their contribution to the domestication of cotton. In general I think the work is interesting, but is probably better suited to a journal with a more specialized readership.

The number one thing I think needs to be addressed here are the issues brought up by Fraser (2019) considering the appearance of compensatory cis trans differences and stabilizing selection. Unless the authors estimate cis and trans from separate replicates, this shouldn't be part of their conclusions. To be clear, I don't require that they do this, only that compensatory changes/stabilizing selection are not considered a conclusion from this work.

> We thank the reviewer for this suggestion. Using the "cross-replicate comparison" method proposed by Fraser (2019), we re-analyzed the *cis* and *trans* effects from separate replicates and showed that the *cis* and *trans* effects were negatively correlated (Pearson's r = -0.29 to -0.34). Although these correlations were much weaker than those estimated by the standard method we previously used (r = -0.78 to -0.80), the negative correlation between *cis* and *trans* effects remains significant, supporting the conclusion that *cis* and *trans* effects often act in opposition.
>
> Other analyses including |*cis*|/(|*cis*|+|*trans*|, |*cis*| versus |*trans*|, and correlations of *cis* or *trans* were also analyzed using the Fraser (2019) method, with results provided in a new supplemental Table S4. Overall, new results are highly consistent with our earlier submission. We hope that this addresses this important concern.

The abstract is hard to follow. Line 20, QTLs are mentioned out of nowhere. Line 23, why do you propose that? Do non-polyploids have less trans regulatory contribution? What are you comparing to?

> We have substantially edited the abstract to improve clarity.

56 I don't know that that logic follows..
70-80 The logic here doesn't really flow. 1. How to estimate cis/trans 2. In other species cis becomes more dominant with time 3. In plants both seem to be important, also in response to environment? 4. How humans effect cis and trans is not explored

> With apologies, we do not understand what the reviewer means exactly, but we made some changes for clarity.

105 discern and parse?

> Changed to "distinguish".

118 what does dpa stand for? Ah, I see it is brought up on line 141, maybe correct that

“(10 and 20 dpa)” was deleted here.

169 As aforementioned

We have modified this sentence according to the new analysis using the Fraser (2019) method, as requested and discussed above.

The use of categories is not very helpful, rather than expecting the reader to remember what category II is, or saying category II (trans only) why not just say what they are – ‘trans only’ ‘cis only’ etc. Again the point here around line 209 and the appearance of compensatory evolution. Also the use of A and B is confusing, just say what they stand for rather than creating a variable, its unnecessary.

We agree, and have mostly replaced the roman numerals with actual categorical terms throughout the manuscript. But for abbreviation and easier cross-reference between figures/tables and the main text, we kept the use of the roman numerals and the ***A***/***B*** metrics, which is also consistent with previous ASE literature.

262 Yoo and Wendel? Maybe just previous work. Also, what is the significance of this?

Replaced by “a previous study”.

276 What is an RD gene

RD is the acronym for “regulatory divergence”, and RD genes refer to the genes that exhibited regulatory divergence consistently between MxT and TxM. This definition was introduced earlier in Results. For added clarity, we modified the sentence inside the parenthesis to be “(i.e., one or both homoeologs belong to the list of 1,655 RD genes; see above)”.

271-282 Not totally sure what you trying to say here – that homeologs with expression differences in wild cotton were more likely to show additional divergence during domestication? Maybe say that more explicitly, or if I’m wrong, state what you are trying to say more clearly.

As suggested, we edited this paragraph to state our finding more explicitly. That is, “fiber genes that already exhibited homoeolog expression bias in wild cotton were more likely to be modified during domestication”.

The whole section on enrichment is speculative. Line 358 – Why? Wouldn’t cis genes be connected in trans to other genes, or are you presuming that cis genes are at the edge of networks? Are these subnetwork density differences significant? What is the maximum density, .012 versus .008 seems like a small difference. Line 362 I don’t understand this sentence.

The reviewer is correct that genes with *cis* variation can also act in *trans* to other genes, so we deleted the sentence that predicted more network connections in *trans* than *cis* genes. Since our results showed that *trans*-only genes with high density are more

interconnected than are *cis*-only genes with low density, we next provided a possible explanation that "these *trans* variations may represent genetic or expression changes of a small number of common upstream regulators, so that *trans* affected genes are more functionally associated and interconnected than are the *cis*-only genes, whose *cis* variations are relatively independent."

Regarding the actual values of density, the differences are apparent given the overall, RD, and non-RD network density shown in Table S7. For example, in TX2094, the overall and non-RD density is 0.006, *cis*-only is 0.008, *trans*-only is 0.013, *cis*+*trans* is 0.011. We inserted the table reference and rewrote L362 as "Interestingly, the density of *cis*+*trans* genes was similar to that of *cis*-only genes in TX2094, but in Maxxa it was more similar to the *trans*-only genes (Table S7; 0.018 in TX2094 and 0.011 in Maxxa)".

Functional enrichment analysis – can we get some p values?

Yes. We specified that all reported enrichment met the criterion of adjusted P <0.05, and all actual P values are provided in Figure S5 and Table S8.

Why was an acronym introduced in that section (GSEA) Will it ever be used again

We deleted this acronym.

385 Is it really necessary to introduce three new acronyms

We believe that the acronyms of GRN (gene regulatory network), TG (transcription factor target) and TF (transcription factor) are necessary abbreviations for describing the complex results of the regulatory gene network analysis.

394 How is this measured? Is it just eye balling? Why do you say smaller gene islands i.e. components? What does that communicate?

In graph theory, a component is defined as a subgraph with each pair of nodes connected by a path. This specific term was used to describe the networks shown in figure 5b (divided into 4 components disconnected from each other) and 5c (a large component that fills most of the network). As pointed out, this term likely causes confusion for readers less familiar with graph theory, so we deleted it from the sentence.

~425 The discussion of network changes is hard to follow. A summary of conclusions and a reference to the figure may be more welcome.

We substantially edited this paragraph to improve clarity.

432 What three fiber QTL genes? Is this from a different paper? Why are QTLs mentioned in the abstract, and then randomly in the results, when you are comparing to a different study? This needs to be clarified. In general the paper is really long and detailed, while also not being clearly written. If you want to compare to QTLs from previous papers, have a section that does this and

clearly summarize the results of overlap between previous QTLs and this study. Were 45 QTLs identified, and 7 overlap with this study?

> In comparison with a previous study (Grover et al 2019[62]), we first stated in the results section that "188 RD genes were identified as candidate fiber domestication genes from a recent QTL study of Maxxa versus TX2094 [62], representing a significant overlap between these two gene lists (Fisher's exact test $P < 0.05$; Figure 5a and Table S4 column "fiber QTL genes")". In the next Results section (GRN analysis), 3 genes out of the list of 53 RD TFs overlapped with the QTL study and are further described. Since these relatively straight-forward results do not require a separate results section, we edited these two sentences in place to improve clarity.

In the discussion, again the point about stabilizing selection.

> Discussion on the new analysis with the Fraser method and other mechanisms underlying compensatory *cis* and *trans* changes is provided in pages 17-18.

In the discussion I think there should be a more succinct summary of the importance of the results, rather than a laundry list of the results again.

> We have shortened the Discussion by removing several sections of redundant results, as suggested.

Again about the categories, why?
529. "the significant correspondence between cis only genes (category I) and additivity by inheritance was expected, given that in this regulatory category, parental expression ratios were maintained between alleles in the F1 hybrids; hence, the aggregated expression of alleles should be intermediate to the parental values unless there exists some form of additional regulatory perturbation. In the case of expression-level dominance whereby the hybrid resembles one of two parental states, significant correspondence was found for trans only genes (category II), as well as those exhibiting compensating effects of coexisting cis and trans variants (IV)."

> We deleted these sentences.

The manuscript is really long and difficult to read.

> As noted by reviewer 1, *cis-trans* analyses in an allopolyploid is complicated conceptually, and accordingly it does not lend itself to brief reports. Our goal here is clarity. As noted throughout our responses, we have shortened the manuscript here and there, without risking lack of clarity or elimination of substantive content. We hope that our revision satisfies the concern of this reviewer.

618 – how is this relevant to the current study? This whole paragraph reads like a book report rather than a summary of previous literature, and how the current study adds to that understanding.

We added the following sentence to wrap up this paragraph: “These examples shed light on how integrated analysis from various data sources might lead to validation of candidate RD genes”

619- Was it enriched for QTL regions? Where was this analysis? I saw one mention of QTLs overlapping at the end of a section, and there was no measure of significant enrichment??

Yes, the significant enrichment result was described in the Results on page 14.

681 Its not the BH method unless you define that acronym, and again why would you, it's the Benjamini-Hochberg method.

Done.

You define CRE in the manuscript as an acronym but never use it, ASE but you only use it one more time after defining it. GRN is redefined twice and not used that many times, GSEA is used twice and defined twice (at the same time). If you want your reader to take the effort of remembering an acronym then use them wisely.

We thank the reviewer for bringing to our attention these issues with abbreviations. We deleted CRE/GSEA and clarified the use of ASE and GRN throughout the text.

It seems like the difference between the two genomes in size should be really interested, and more fully explored. The paper is really long and detailed, and I’m not sure how interesting or useful a lot of the information provided is. Just because an analysis was done, does not mean it needs to be included in the paper.

We agree that the difference in genome size is fascinating, likely important, and helped motivate the experiments described in this manuscript. Allopolyploidy is so very common in plants, and yet we still understand very little about how selection and evolution in an allopolyploid differ from that in a diploid. We find this to be very interesting, and hope that this clearer revision will make this point evident to the reviewer. We also draw attention to the subgenome size difference more on page 19: “It is worth noting that different parental TE loads and their relative distribution between subgenomes have been the most popular explanation for biased homoeolog expression and biased genome fractionation [104, 105, 106, 107], which likely further complicates regulatory circuits in allopolyploids.”

Figure 2A What is the difference between compensating and compensatory? Why can you only see red, blue, purple, and maroon in these figures? If the colors apply to a, not b, and then c-f you might consider reordering the figures

We have modified Figure 2 as suggested. The color legend was moved to the left and grouped by parental divergence $\boldsymbol{A} \neq 0$ and $\boldsymbol{A} = 0$: Figure 2a showed the relative proportions of $\boldsymbol{A} \neq 0$ categories (red, blue, purple, and maroon) and Figure 2c-f showed the boxplots for all seven categories.

Reviewer #3:

Review of Bao et al., Unraveling cis and trans regulatory evolution during cotton domestication.

Overview:

In this manuscript, the authors undertake a large-scale genetic cross/RNA-seq analysis to understand the nature of the regulatory divergence between wild and domesticated cotton. They find large contributions to differential expression from both cis and trans changes, often finding that the changes are in compensating directions, such that overall expression level is conserved across the strains.

Major Comments:
This is a well-executed and interesting experiment that adds to our understanding of regulatory evolution. However, I have some reservations about the statistical analyses and presentation that I think lead the manuscript to be overly definitive in its interpretation of the data collected.

My first concern is best understood when comparing class IV and V in figure 2: The "compensatory" class represents parents with no observed differential expression yet showing allele-specific differences in the hybrids. The issue this raises is that the statistical models the authors use to identify differentially-expressed genes are perfectly valid, but are intrinsically pair-wise. Hence, effects such as differences in statistical power can give rise to cases such #V, which arguably could be an instance where the difference between the parents was too slight to reject the null hypothesis but for some reason exceeded that threshold in the F1. Although I have given this as a particular example, I think the problem is general: strictly assigning genes or alleles as either differentially-expressed or not in pairwise comparisons and then using those test results to build the sort of cis-trans model used here is problematic because the inherent variation in the experiment is only considered in the pairwise comparisons, but there is also a component of variation between the parents and the F1s, such that even in a "perfect" cis case we do not expect the parental expression levels to precisely predict the F1 levels. Perhaps the authors' approach captures this problem, but it does not appear so from the methods: instead, the authors assign the part of the variance not explained by cis effects in the F1 as by definition trans effects. But the t-test used to do this, would not, to my mind, capture the fact that there are unmeasured errors in the estimates of the size of the cis effects in both parents and in the F1s. Hence this "trans" category represents true trans effects as well as simple experimental noise not captured by the authors' statistical approaches. I would strongly urge the authors to consult a statistician, if for no other reason than to refute the argument above.

> We deeply appreciate this very thoughtful comment (this is a perfect example of how the revision process should work!). As recently discussed by Fraser (Trends in Genetics, 2019), the current method for estimating *cis* and *trans* effects in ASE analysis is intrinsically biased to overestimate the negative correlations between *cis* and *trans* effects. The cause of this bias is indeed the "pairwise" (more precisely to say "coupled") inference of *cis* and *trans*, just as the reviewer pointed out: any error in estimating *cis* will be passed onto the *trans* estimates, hence introducing an artifactual negative correlation between them.

One solution to this bias, as proposed by Fraser (2019), is using different biological replicates to estimate *cis* and *trans* separately, which keeps errors uncoupled and random with respect to each other. More specifically, one replicate can be used to estimate the *cis* effect ***B***, and a different replicate needs to be used to calculate ***B'*** for estimating the *trans* effect ***A – B'***. Applying this cross-replicate approach to our data, we analyzed all six possible combinations of 3 biological replicates and obtained the correlations between *cis* and *trans* - Pearson's r = -0.29 to -0.34. These negative correlations are weaker than those derived from the standard approach (all 3 replicates used by both *cis* and *trans* estimates; r = -0.78 to -0.80); however, they remain significant. Thus, the important conclusion from our original manuscript still holds; that is, *cis* and *trans* effects often act in opposition. This new analysis based on Fraser (2019), and relevant discussion, has been added to the revised manuscript.

Regarding our categorization results, we would like to clarify that our method indeed captures this problem of *cis* and *trans* estimates from the standard approach. As illustrated in Figure 1A, our analysis relies on the variation tests of parental and allelic expression difference (***A***, ***B***, and ***A***vs***B***) to classify genes into distinct categories of regulatory evolution, rather than the actual estimates of *cis* and *trans*. The use of significance cutoff ($P < 0.05$) is expected to eliminate noise to a reasonable extent (e.g. misplace a highly variable ***B*** as true *cis*, or a highly variable ***A***vs***B*** as true *trans*); in addition, those combinations of conflicting variation test results were assigned to the "Ambiguous" category. We also tested a series of different cutoffs in stringency, which led to consistent patterns, as described in Results.

The reviewer mentioned that "even in a 'perfect' *cis* case we do not expect the parental expression levels to precisely predict the F1 levels." This is absolutely correct, because it is the pre-assumption of ASE analysis that in a synthetic F1 allelic expression reflects only *cis* divergence between the parents, but as we know and point out, this assumption is by no means guaranteed. In this revision, we further discussed this important caveat of ASE analysis, as well as the alternative and complementary eQTL analysis possible in future work in pages 17-18.

A related concern that I don't think requires more analysis but needs to be at least acknowledged by the authors is that the model used assumes no interactions between cis and trans effects, allowing the separation described above. Now we know that there *are* interactions: from a simple biochemical perspective, if one cis binding site increases its affinity for a trans-factor, the excess binding of that factor to that site decreases the apparent concentration of the factor at the other allele, even if the overall concentration of the factor is unaltered. This is only an example: there are multiple ways in which biochemical systems feedback and adapt. Hence, while I think the authors' approach is acceptable for a first-pass analysis, I think the assumption on which the cis/trans differentiation is being made should be highlighted more.

The reviewer is correct in pointing out this complexity, one of so very many that potentially impact the interaction of *cis* and *trans* function and hence evolution. In an effort to acknowledge this without adding more length to the manuscript (noting the

concern about length raised by others), we mention this in the introduction, citing Hu et al. (2019) and Veitia et al. (2018), recent papers in which some of these complexities are illuminated for allopolyploids: "Understanding *cis and trans*-interactions in an allopolyploid is not only made more complicated by the presence of duplicated suites of interacting factors, but by the fact that even in a diploid regulatory interactions are subject to many different forms of biochemical and stoichiometric control and feedbacks [57, 58]".

These interaction effects lead me to my final major concern: the authors' interpretation of the excess of compensating cis/trans interactions. The authors attribute the excess of compensating interactions over enhancing ones to the actions of purifying selection on gene expression levels: presumably through the fixation of compensating cis changes after a trans change or vice versa. This is a possible interpretation that is likely correct in some cases. But it ignores a very plausible null hypothesis, which is that these regulatory systems are subject to various forms of feedback (1). Feedback regulation at the mRNA or protein level will produce this same excess of compensatory changes in expression levels without a need to directly invoke selection on expression levels (2). Moreover, the feedback regulation need not be due to direct selection on expression level: it can also result from selection against noise in gene expression (3).

Very true, and we thank the reviewer for this comment. We have added the feedback mechanism in the discussion on pages 17-18.

There are a few more minor issues with the manuscript, but I think they can more appropriately be addressed once the overall framework of the manuscript is improved with better statistical models.

We have thoroughly edited this manuscript according to all reviewers' comments, and hope this revision meet the expectations of the reviewers.

References:
1. Becskei A & Serrano L (2000) Engineering stability in gene networks by autoregulation. Nature 405(6786):590-593.
2. Wagner A (2005) Robustness and Evolvability in Living Systems (Princeton University Press, Princeton, NJ).
3. Rao CV, Wolf DM, & Arkin AP (2002) Control, exploitation and tolerance of intracellular noise. Nature420(6912):231-237.

Citations added.

REVIEWERS' COMMENTS:

Reviewer #1 (Remarks to the Author):

The authors have addressed all of my concerns and corrected the errors and confusing sentences I identified in the original submission. The paper reads more clearly to me now (perhaps also because I've read it a couple of additional times!). It describes some very interesting original work, as I stated in my previous review, and I believe it will be a solid contribution to the literature on gene regulatory evolution in allopolyploids, with relevance to domestication (though see my comments, below, about generalizing from particular genotypes to species and the process of domestication).

Specific comments (all of a minor or relatively minor nature), by line number:

214. inheritance in in those genes (delete one "in")

335. Dt promoters accumulated more SNPs than did At promoters

I tried to ascertain exactly what was meant here. I believe that the more precise terminology would be "promoter regions" or better still, "5' noncoding regions within 2 kb of transcription start sites of annotated genes." It is possible that the precise sequences of promoters for all of these genes have been identified and validated functionally … but I doubt it. I referred to Methods to see what was done for this section, and had to look up SnpEff (this is not a tool I have used) to try to see what it does. My conclusion was that it does not give the information such as would be provided by MEME, using lists of known TF binding sites, etc. I could be wrong, however.

352-355. suggesting that regulatory changes under domestication were biased toward targeting genes that were highly connected in wild fiber developmental networks. In contrast, these RD genes were not enriched as hubs in the Maxxa network, likely reflecting the alteration of fiber network topology by domestication.

I found this difficult. It is only the wild species' network that is significantly enriched for RD genes. I don't see a clear connection between this and domestication-induced changes. Why would the Maxxa network not also show enrichment for genes showing regulatory divergence? I'm confident that the authors have an interesting point to make here, but I think a bit more of the logic needs to be laid out.

448-450. 15% of the genes in the fiber transcriptome have experienced some form of regulatory alteration between wild and domesticated G. hirsutum accessions.

What about standing variation in the wild species? Perhaps change to "... differ in their regulatory pattern between this pair of wild and domesticated ... "?

451. wild and domesticated G. hirsutum

Add TX... and Maxxa names here to help readers who jump to Discussion without reading earlier parts and thus will not know the accession names.

465-466. changes implicated by domestication

Should be "implicated in"

469. are differentially expressed between wild and domesticated cotton

As noted in a previous comment, all of these comparisons involve individuals from single accessions of

wild and domesticated cottons. I believe that the authors should be circumspect in claiming that the changes they document so carefully truly represent all members of wild and domesticated cotton. This is particularly true for the wild species. In this instance, I would suggest something like, "… between these wild and domesticated accessions".

488. correlation, are not entirely

Delete comma.

491. and intraspecific studies, whereas exceptions are common

Change "whereas" to "but".

504-505. the number of cis vs. trans regulated alleles between these two … accessions is nearly equal, in contrast to most evidence from other plant … systems.

AND

521-523. This phenomenon … may be a general and previously unrecognized feature of polyploidy, perhaps helping to explain evolutionary novelty in allopolyploid plants

These two statements contradict one another. All plants are fundamentally polyploid, so "most evidence" from plants necessarily refers to polyploids. Thus, although I find parts of this hypothesis plausible and interesting, it cannot explain regulatory evolution in polyploids if "most evidence" contradicts it. Perhaps the authors intend it to only refer to early stages in polyploid plant evolution? If so, this needs to be made more clear.

557-558. Under domestication, regulatory changes of 15% of individual fiber expressed genes

This is another instance of the problem referred to in my comments on line 469. It is fair to say that these differences are between a domesticated and a wild accession (genotype), but not that they occurred as a direct consequence of domestication, despite the likelihood that this is the case.

Jeff Doyle

Reviewer #2 (Remarks to the Author):

I do not think that this is the most clearly written manuscript, however I do not believe there are methodological problems that remain to be addressed. If the editors find that it is suitable for publication, I have no objection.

Reviewer #3 (Remarks to the Author):

The authors have addressed my core concerns, and I have no further comments.

Reviewer #1:

The authors have addressed all of my concerns and corrected the errors and confusing sentences I identified in the original submission. The paper reads more clearly to me now (perhaps also because I've read it a couple of additional times!). It describes some very interesting original work, as I stated in my previous review, and I believe it will be a solid contribution to the literature on gene regulatory evolution in allopolyploids, with relevance to domestication (though see my comments, below, about generalizing from particular genotypes to species and the process of domestication).

Specific comments (all of a minor or relatively minor nature), by line number:

214. inheritance in in those genes (delete one "in")

Done.

335. Dt promoters accumulated more SNPs than did At promoters

I tried to ascertain exactly what was meant here. I believe that the more precise terminology would be "promoter regions" or better still, "5' noncoding regions within 2 kb of transcription start sites of annotated genes." It is possible that the precise sequences of promoters for all of these genes have been identified and validated functionally … but I doubt it. I referred to Methods to see what was done for this section, and had to look up SnpEff (this is not a tool I have used) to try to see what it does. My conclusion was that it does not give the information such as would be provided by MEME, using lists of known TF binding sites, etc. I could be wrong, however.

We edited the text in parenthesis as suggested: "6.4 vs. 5.7 SNPs on average within 2 Kb upstream of the annotated transcription start sites".

352-355. suggesting that regulatory changes under domestication were biased toward targeting genes that were highly connected in wild fiber developmental networks. In contrast, these RD genes were not enriched as hubs in the Maxxa network, likely reflecting the alteration of fiber network topology by domestication.

I found this difficult. It is only the wild species' network that is significantly enriched for RD genes. I don't see a clear connection between this and domestication-induced changes. Why would the Maxxa network not also show enrichment for genes showing regulatory divergence? I'm confident that the authors have an interesting point to make here, but I think a bit more of the logic needs to be laid out.

We edited the second sentence as: "Likely as a result of these regulatory changes, the network properties of these RD genes were altered and found not enriched as hubs in the Maxxa network".

448-450. 15% of the genes in the fiber transcriptome have experienced some form of regulatory alteration between wild and domesticated G. hirsutum accessions.

What about standing variation in the wild species? Perhaps change to "... differ in their regulatory pattern between this pair of wild and domesticated ... "?

Agreed; we edited the sentence accordingly.

451. wild and domesticated G. hirsutum

Add TX... and Maxxa names here to help readers who jump to Discussion without reading earlier parts and thus will not know the accession names.

Done.

465-466. changes implicated by domestication

Should be “implicated in”

Done.

469. are differentially expressed between wild and domesticated cotton

As noted in a previous comment, all of these comparisons involve individuals from single accessions of wild and domesticated cottons. I believe that the authors should be circumspect in claiming that the changes they document so carefully truly represent all members of wild and domesticated cotton. This is particularly true for the wild species. In this instance, I would suggest something like, “… between these wild and domesticated accessions”.

We have made edits throughout the text as suggested.

488. correlation, are not entirely

Delete comma.

Deleted.

491. and intraspecific studies, whereas exceptions are common

Change “whereas” to “but”.

Changed.

504-505. the number of cis vs. trans regulated alleles between these two … accessions is nearly equal, in contrast to most evidence from other plant … systems.

AND

521-523. This phenomenon … may be a general and previously unrecognized feature of polyploidy, perhaps helping to explain evolutionary novelty in allopolyploid plants

These two statements contradict one another. All plants are fundamentally polyploid, so “most evidence” from plants necessarily refers to polyploids. Thus, although I find parts of this hypothesis plausible and interesting, it cannot explain regulatory evolution in polyploids if “most evidence” contradicts it. Perhaps the authors intend it to only refer to early stages in polyploid plant evolution? If so, this needs to be made more clear.

> Thank you for pointing out this confusion. We edited the paragraph to make it clear that the cotton results are in contrast to evidence from some other systems such as maize, and our discussion refers to recently formed allopolyploid plants.

557-558. Under domestication, regulatory changes of 15% of individual fiber expressed genes

This is another instance of the problem referred to in my comments on line 469. It is fair to say that these differences are between a domesticated and a wild accession (genotype), but not that they occurred as a direct consequence of domestication, despite the likelihood that this is the case.

Jeff Doyle

> Agreed, and we have made edits throughout the text as suggested.

Reviewer #2

I do not think that this is the most clearly written manuscript, however I do not believe there are methodological problems that remain to be addressed. If the editors find that it is suitable for publication, I have no objection.

Reviewer #3

The authors have addressed my core concerns, and I have no further comments.

> We thank all reviewers for their excellent comments and criticisms, which prompted us to revise the manuscript for improved accuracy, robustness and clarity.
